# Supplementary material for: A Distance-Dependent Distribution of Presynaptic Boutons Tunes Frequency-Dependent Dendritic Integration
Source: Neuron. 2018 Jul 25;99(2):275–282.e3. doi: 10.1016/j.neuron.2018.06.015 (PMC6078905; doi:10.1016/j.neuron.2018.06.015)
Supplement: Document S1. Figures S1–S4 [file mmc1.pdf]

Neuron, Volume *99*

Supplemental Information

A Distance-Dependent Distribution of Presynaptic Boutons Tunes Frequency-Dependent Dendritic Integration

Federico W. Grillo, Guilherme Neves, Alison Walker, Gema Vizcay-Barrena, Roland A. Fleck, Tiago Branco, and Juan Burrone

Figure S1

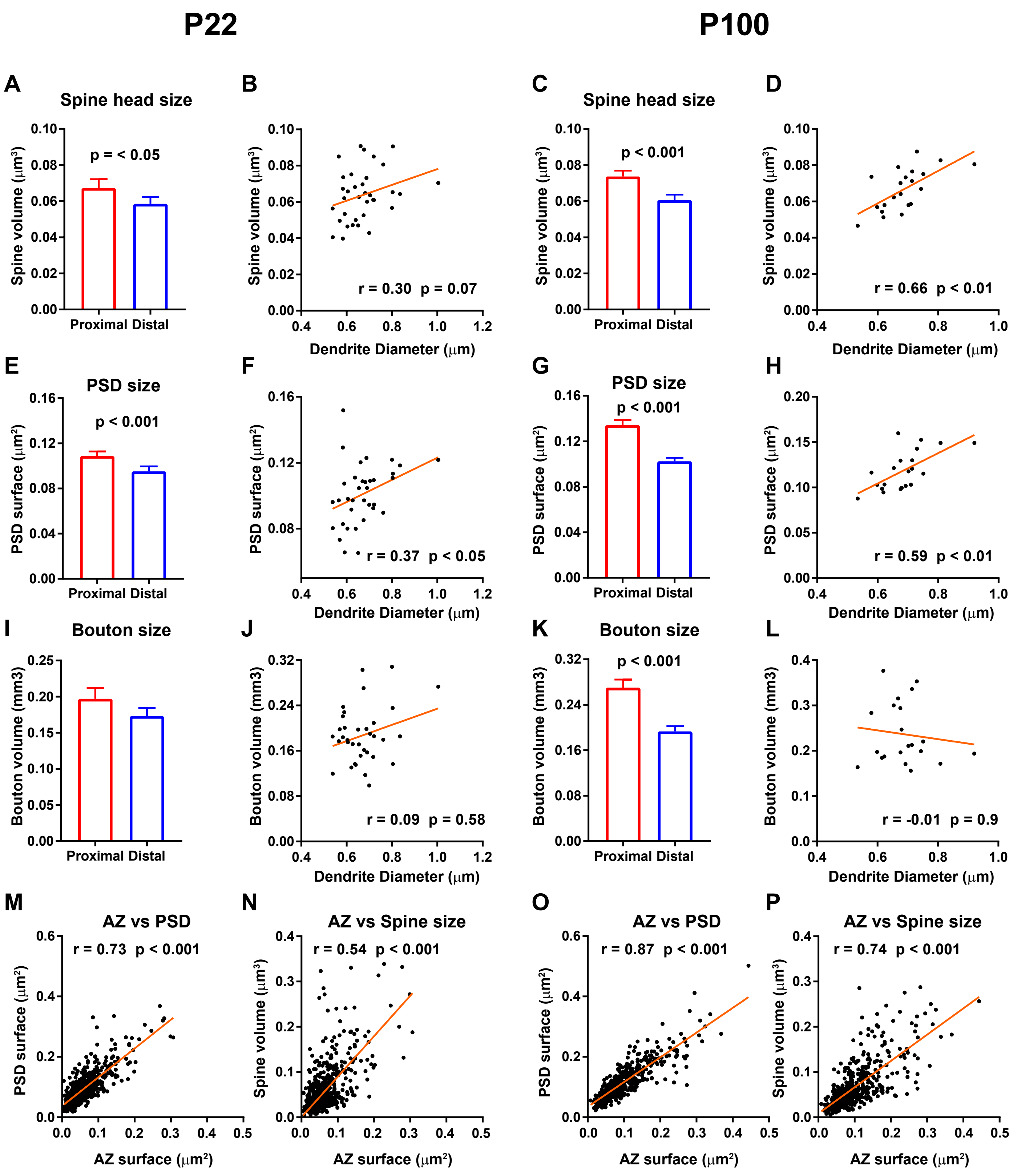
P22
P100
A
Spine head size
Spine volume (µm³)
p = < 0.05
Proximal Distal
B
Spine volume (µm³)
r = 0.30 p = 0.07
Dendrite Diameter (µm)
C
Spine head size
Spine volume (µm³)
p < 0.001
Proximal Distal
D
Spine volume (µm³)
r = 0.66 p < 0.01
Dendrite Diameter (µm)
E
PSD size
PSD surface (µm²)
p < 0.001
Proximal Distal
F
PSD surface (µm²)
r = 0.37 p < 0.05
Dendrite Diameter (µm)
G
PSD size
PSD surface (µm²)
p < 0.001
Proximal Distal
H
PSD surface (µm²)
r = 0.59 p < 0.01
Dendrite Diameter (µm)
I
Bouton size
Bouton volume (mm3)
Proximal Distal
J
Bouton volume (mm3)
r = 0.09 p = 0.58
Dendrite Diameter (µm)
K
Bouton size
Bouton volume (mm3)
p < 0.001
Proximal Distal
L
Bouton volume (mm3)
r = -0.01 p = 0.9
Dendrite Diameter (µm)
M
AZ vs PSD
r = 0.73 p < 0.001
PSD surface (µm²)
AZ surface (µm²)
N
AZ vs Spine size
r = 0.54 p < 0.001
Spine volume (µm³)
AZ surface (µm²)
O
AZ vs PSD
r = 0.87 p < 0.001
PSD surface (µm²)
AZ surface (µm²)
P
AZ vs Spine size
r = 0.74 p < 0.001
Spine volume (µm³)
AZ surface (µm²)

Figure S1. Synaptic properties for P22 and P100 dendrites reconstructed by SBFSEM. Related to Figure 1. For all P22 mice, N = 35 dendrites, 604 synapses. For P100 mice, N = 26 dendrites, 505 synapses. **(A-D)** Postsynaptic spine volume is smaller in distal dendrites compared to proximal dendrites in P22 and P100 mice (Wilkoxon Rank-Sum for A and C; Spearman's correlation for B and D). **(E-H)** Postsynaptic density (PSD) is smaller in distal dendrites compared to proximal dendrites in P22 and P100 mice (Wilkoxon Rank-Sum for E and G; Spearman's correlation for F and H). **(I-L)** Presynaptic bouton size (volume) in distal dendrites compared to proximal dendrites in P22 and P100 mice (Wilkoxon Rank-Sum for I and K; Spearman's correlation for J and L). **(M-P)** Correlations between PSD and AZ size (M and O, Spearman's correlation) and between spine volume and AZ size (N and P, Spearman's correlation) in P22 and P100 mice.

Figure S2

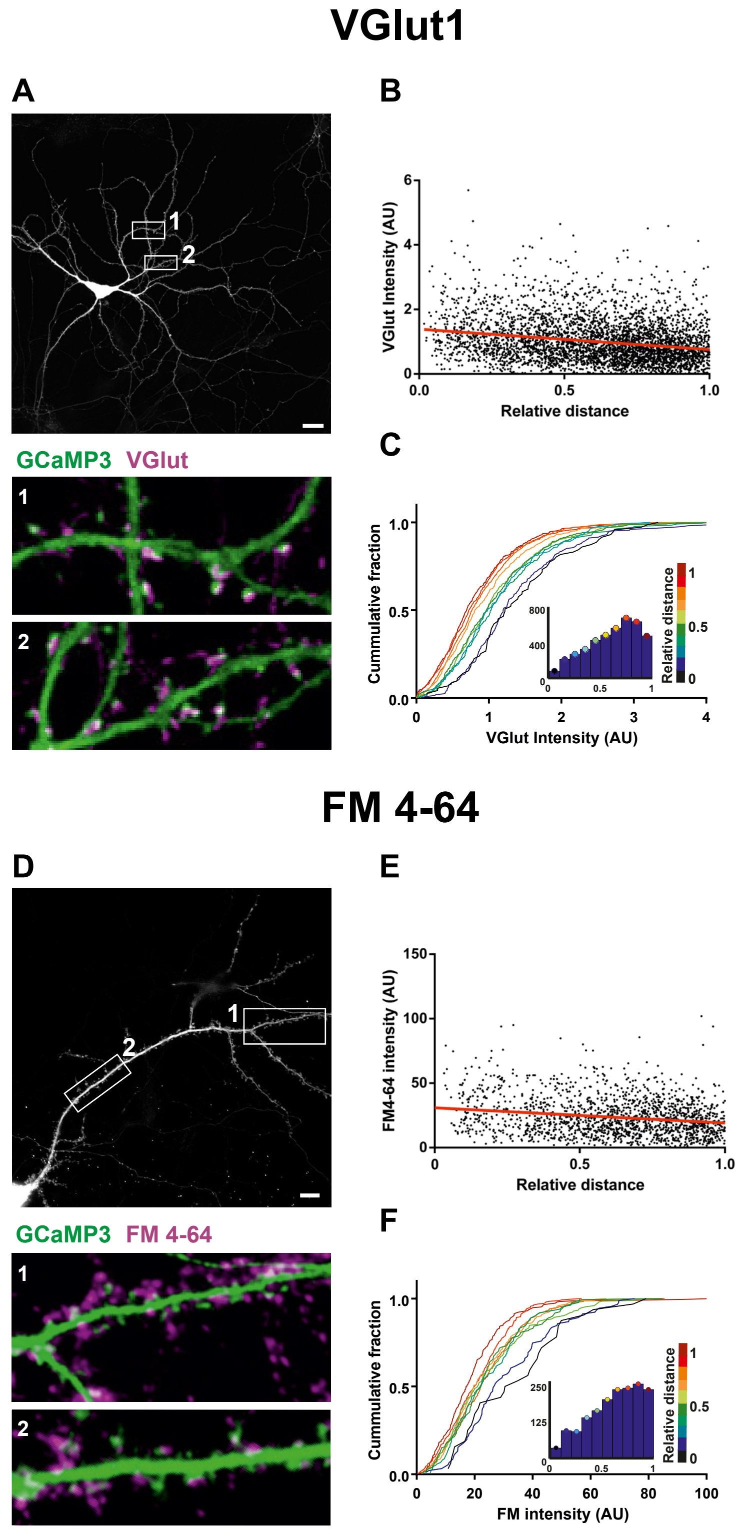
VGlut1
A
B
1
2
GCaMP3 VGlut
1
2
VGlut Intensity (AU)
6
4
2
0
0.0
0.5
1.0
Relative distance
C
Cummulative fraction
1.0
0.5
0.0
0
1
2
3
4
VGlut Intensity (AU)
800
400
0
0
0.5
1
Relative distance
1
0.5
0
FM 4-64
D
E
1
2
GCaMP3 FM 4-64
1
2
FM4-64 intensity (AU)
150
100
50
0
0
0.5
1.0
Relative distance
F
Cummulative fraction
1.0
0.5
0.0
0
20
40
60
80
100
FM intensity (AU)
250
125
0
0
0.5
1
Relative distance
1
0.5
0

Figure S2. Preynaptic structure and function scales inversely with relative distance along a dendrite in dissociated neurons. Related to Figure 2. (A-C) Immunocytochemical labelling of vGlut1. **(A)** Example hippocampal neurons expressing GCaMP3, used here as a cytoplasmic marker. White box corresponds to the areas shown in the zoomed region below, numbered appropriately. Scale bars = 10 µm. Zoomed regions show immunostain (magenta) overlaid onto the GCaMP3 expressing cell (green). **(B)** vGlut immunostain intensity for each puncta plotted as a function of relative dendritic distance to the soma. Spearman's correlation ($P<0.0001$, $R^2=0.94$, n=4051 from 4 cells). **(C)** Cumulative frequency distributions for immunostain intensity for each pucta where each plot is colour-matched to a different bin for relative distance shown in the key to the right. Inset histograms show the number of spines included within each relative distance bin. **(D-F)** Labelling of vesicle recylcling with FM4-64. **(D)** Example hippocampal neurons expressing GCaMP3. White box corresponds to the areas shown in the zoomed region below, numbered appropriately. Scale bars = 10 µm. Zoomed regions show FM4-64 stain (magenta) overlaid onto the GCaMP3 expressing cell (green). **(E)** FM4-64 label intensity for each puncta plotted as a function of relative dendritic distance to the soma. Spearman's correlation ($P=0.001$, $R^2=0.85$, n=1650 spines from 7 cells). **(F)** Cumulative frequency distributions for FM4-64 intensity for each pucta where each plot is colour-matched to a different bin for relative distance shown in the key to the right. Inset histograms show the number of spines included within each relative distance bin. Relative dendritic distance was calculated as the absolute distance (shortest path to the soma) divided by the distance from the soma to the furthest branch tip, calculated using an algorithm that searches for the longest path from each ROI to a branch tip.

Figure S3

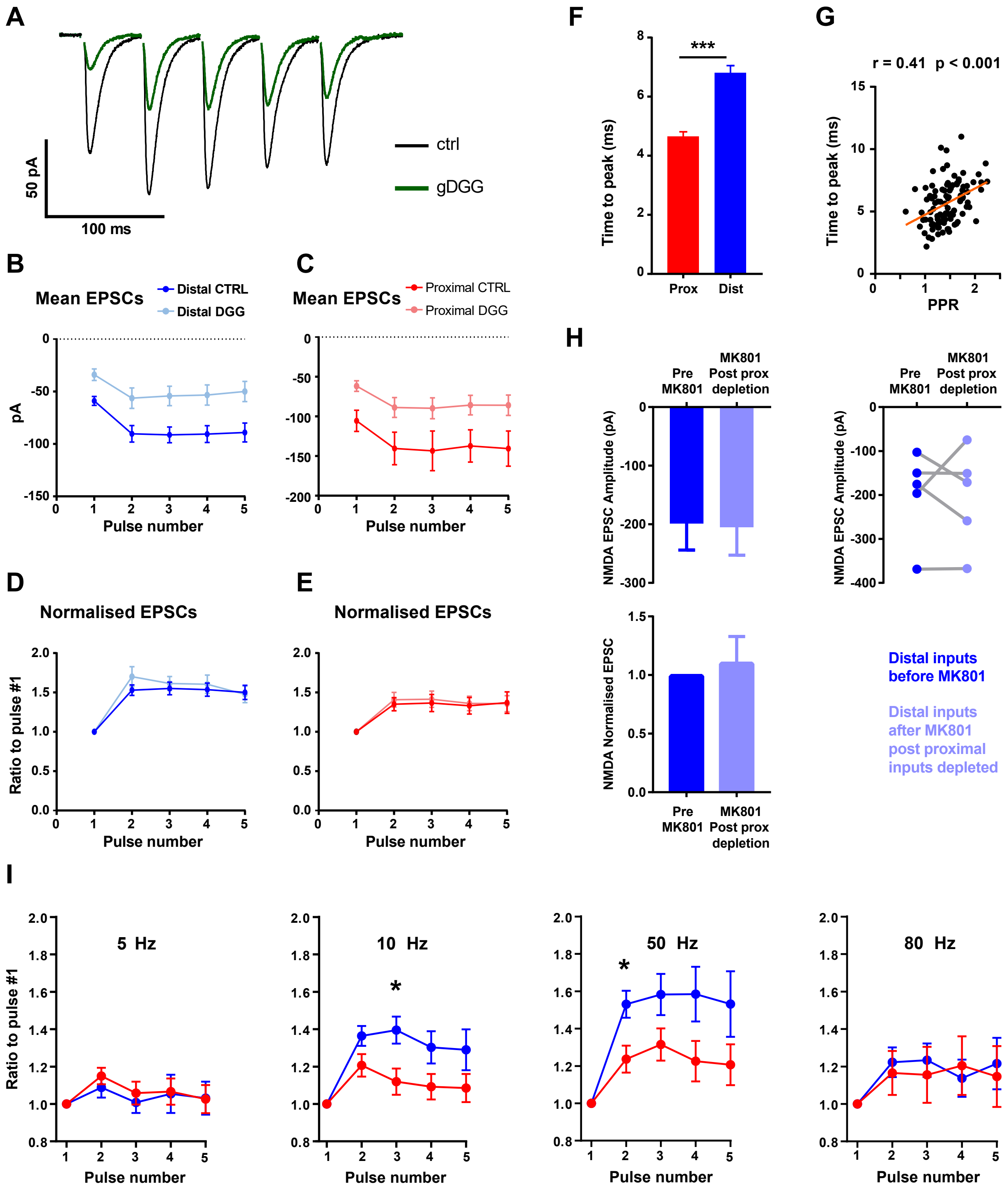
A
ctrl
gDGG
50 pA
100 ms
B
Mean EPSCs
Distal CTRL
Distal DGG
pA
Pulse number
C
Mean EPSCs
Proximal CTRL
Proximal DGG
Pulse number
D
Normalised EPSCs
Ratio to pulse #1
Pulse number
E
Normalised EPSCs
Pulse number
F
***
Time to peak (ms)
Prox
Dist
G
r = 0.41 p < 0.001
Time to peak (ms)
PPR
H
Pre MK801
MK801 Post prox depletion
NMDA EPSC Amplitude (pA)
NMDA Normalised EPSC
Distal inputs before MK801
Distal inputs after MK801 post proximal inputs depleted
I
5 Hz
10 Hz
50 Hz
80 Hz
*
Ratio to pulse #1
Pulse number

Figure S3. **Physiological properties of proximal and distal synapses. Related to Figure 2.** γ-D-Glutamylglycine (γDGG) is an AMPA glutamate receptor antagonist with low affinity and fast dissociation kinetics. When present in the millimolar range it competes with released glutamate causing a reduction of AMPAR current. **(A)** EPSC amplitude is reduced, compared to control (black trace), following γDGG application (gray trace). **(B-C)** Mean 5 pulse stimulus response amplitudes before and during 1.5 mM γDGG bath application. **(D-E)** Normalised EPSCs for 5 pulse trains before and during 1.5 mM γDGG bath application. Although not significant ($P = 0.15$, two-tailed paired t test) there is a small increase in PPR that is more marked in distal **(D)** compared to proximal synapses **(E)** and may reflect an increased occurrence of multi vesicular release between the first and second pulse. **(F)** The time to peak of an EPSC was significantly higher in distally triggered events (49 cells, mean = prox 6.8 ± 0.24 ms, dist 4.65 ± 0.16 ms, $p < 0.0001$ Wilcoxon sign rank test) with longer times correlating to the amount of facilitation **(G)**, Spearman's correlation. **(H)** Distal NMDAR mediated responses are not affected following blockade of proximal NMDARs with MK-801, N=5 cells. Top left: average amplitude of EPSCs prior to MK801 bath application (dark blue, average of 20 sweeps) and after proximal inputs have been depleted (First response to firs stimulus) in MK-801 stimulation protocol (Figure 2J) (light blue); top right: changes in the amplitudes of proximal NMDAR responses for individual cells following depletion of distal NMDAR responses (N = 5 cells); bottom left: average NMDAR responses normalized to the response before MK-801 proximal depletion. **(I)** Facilitation over trains of 5 pulses is frequency-dependent. No statistical difference between proximal and distal responses found at 5Hz (n = 13 cells) and 80 Hz (n = 9). At 10 Hz (n = 16) greater distal facilitation was detected at pulse #3, $p < 0.05$; at 50 Hz (n = 20) greater distal facilitation was detected at pulse #2, $p < 0.05$, multiple t tests with Holm-Sidak adjusted p values.

Figure S4

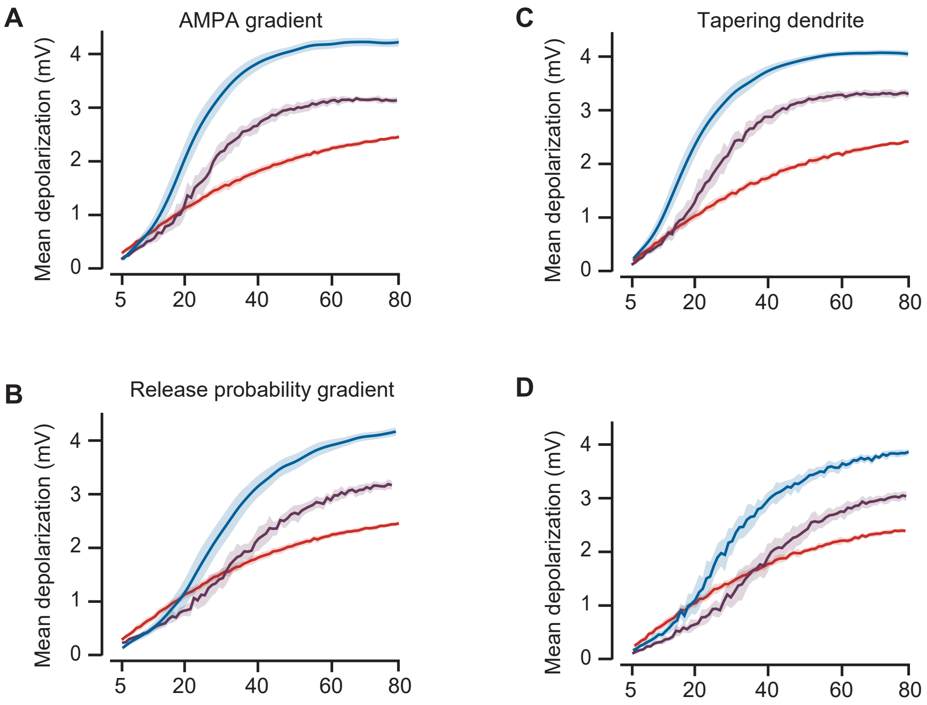

A
AMPA gradient
Mean depolarization (mV)
0
1
2
3
4
5
20
40
60
80
C
Tapering dendrite
Mean depolarization (mV)
0
1
2
3
4
5
20
40
60
80
B
Release probability gradient
Mean depolarization (mV)
0
1
2
3
4
5
20
40
60
80
D
Mean depolarization (mV)
0
1
2
3
4
5
20
40
60
80

Figure S4. PPR gradient enhances distal supra-linear integration in the presence of additional gradients. Related to Figure 3. Input-output curves for models with proximo-distal gradients of **(A)** AMPA receptor conductances (distal = 81% of proximal), **(B)** release probability (distal = 70% of proximal), **(C)** dendrite diameter (distal = 85% of proximal), and **(D)** all three gradients together. For all plots red curves show proximal synapses, blue show distal synapses and purple show distal synapses with proximal PPR. Shaded area is SEM. All values for the gradients used in the model were obtained from measurements taken from Figures 1 and 2.

Supplementary text. Code for the compartmental model used in Figure 3 and S4. Compartmental model of dendritic integration that implements synaptic short-term facilitation and depression as described previously (Varela et al., 1997). Simulations were performed with the NEURON simulation environment (Hines and Carnevale, 1997).
