# Supplementary material for: A Distance-Dependent Distribution of Presynaptic Boutons Tunes Frequency-Dependent Dendritic Integration
Source: Neuron. 2018 Jul 25;99(2):275–282.e3. doi: 10.1016/j.neuron.2018.06.015 (PMC6078905; doi:10.1016/j.neuron.2018.06.015)
Supplement: Data S1. Code for the Compartmental Model Used in Figures 3 and S4 — Compartmental model of dendritic integration that implements synaptic short-term facilitation and depression as described previously (Varela et al., 1997). Simulations were performed with the NEURON simulation environment (Hines and Carnevale, 1997). [file mmc2.pdf]

Supplementary Text. Code for compartmental model used in Figure 3 and Figure S4.

```
COMMENT
Implementation of the model of short-term facilitation and depression
described in
  Varela, J.A., Sen, K., Gibson, J., Fost, J., Abbott, L.R., and Nelson,
S.B.
  A quantitative description of short-term plasticity at excitatory
synapses
  in layer 2/3 of rat primary visual cortex
  Journal of Neuroscience 17:7926-7940, 1997
This is a modification of Exp2Syn that can receive multiple streams of
synaptic input via NetCon objects. Each stream keeps track of its own
weight and activation history.

The printf() statements are for testing purposes only.


The synaptic mechanism itself uses a two state kinetic scheme described
by
rise time tau1 and decay time constant tau2.
The normalized peak condunductance is 1.
Decay time MUST be greater than rise time.

The solution of A->G->bath with rate constants 1/tau1 and 1/tau2 is
 A = a*exp(-t/tau1) and
 G = a*tau2/(tau2-tau1)*(-exp(-t/tau1) + exp(-t/tau2))
      where tau1 < tau2

If tau2-tau1 -> 0 then we have a alphasynapse.
and if tau1 -> 0 then we have just single exponential decay.

The factor is evaluated in the
initial block such that an event of weight 1 generates a
peak conductance of 1.

Because the solution is a sum of exponentials, the
coupled equations can be solved as a pair of independent equations
by the more efficient cnexp method.

ENDCOMMENT

NEURON {
      POINT_PROCESS FDSExp2Syn
      RANGE tau1, tau2, e, i
      NONSPECIFIC_CURRENT i

      RANGE g
      GLOBAL total
        RANGE f, tau_F, d1, tau_D1, d2, tau_D2
}

UNITS {
      (nA) = (nanoamp)
      (mV) = (millivolt)
      (umho) = (micromho)
}

PARAMETER {
      tau1 = 0.1 (ms) < 1e-9, 1e9 >
```

```
      tau2 = 10 (ms) < 1e-9, 1e9 >
      e = 0 (mV)
        : these values are from Fig.3 in Varela et al. 1997
      : the (1) is needed for the range limits to be effective
        f = 0.917 (1) < 0, 1e9 >    : facilitation
        tau_F = 94 (ms) < 1e-9, 1e9 >
        d1 = 0.416 (1) < 0, 1 >     : fast depression
        tau_D1 = 380 (ms) < 1e-9, 1e9 >
        d2 = 0.975 (1) < 0, 1 >     : slow depression
        tau_D2 = 9200 (ms) < 1e-9, 1e9 >
}

ASSIGNED {
      v (mV)
      i  (nA)
      g (umho)
      factor
      total (umho)
}

STATE {
      A (umho)
      B (umho)
}

INITIAL {
      LOCAL tp
      total = 0
      if (tau1/tau2 > 0.9999) {
            tau1 = 0.9999*tau2
      }
      A = 0
      B = 0
      tp = (tau1*tau2)/(tau2 - tau1) * log(tau2/tau1)
      factor = -exp(-tp/tau1) + exp(-tp/tau2)
      factor = 1/factor
}

BREAKPOINT {
      SOLVE state METHOD cnexp
      g = B - A
      i = g*(v - e)
}

DERIVATIVE state {
      A' = -A/tau1
      B' = -B/tau2
}

NET_RECEIVE(weight (umho), F, D1, D2, tsyn (ms)) {
INITIAL {
: these are in NET_RECEIVE to be per-stream
        F = 1
        D1 = 1
        D2 = 1
        tsyn = t
: this header will appear once per stream
: printf("t\t t-tsyn\t F\t D1\t D2\t amp\t newF\t newD1\t newD2\n")
```

```
}

        F = 1 + (F-1)*exp(-(t - tsyn)/tau_F)
        D1 = 1 - (1-D1)*exp(-(t - tsyn)/tau_D1)
        D2 = 1 - (1-D2)*exp(-(t - tsyn)/tau_D2)
: printf("%g\t%g\t%g\t%g\t%g\t%g", t, t-tsyn, F, D1, D2, weight*F*D1*D2)
        tsyn = t

      state_discontinuity(A, A + weight*factor*F*D1*D2)
      state_discontinuity(B, B + weight*factor*F*D1*D2)
      total = total+weight*F*D1*D2

        F = F + f
        D1 = D1 * d1
        D2 = D2 * d2
: printf("\t%g\t%g\t%g\n", F, D1, D2)
}
```

```
COMMENT
Implementation of the model of short-term facilitation and depression
described in
  Varela, J.A., Sen, K., Gibson, J., Fost, J., Abbott, L.R., and Nelson,
S.B.
  A quantitative description of short-term plasticity at excitatory
synapses
  in layer 2/3 of rat primary visual cortex
  Journal of Neuroscience 17:7926-7940, 1997
This is a modification of Exp2Syn that can receive multiple streams of
synaptic input via NetCon objects. Each stream keeps track of its own
weight and activation history.

The printf() statements are for testing purposes only.


The synaptic mechanism itself uses a two state kinetic scheme described
by
rise time tau1 and decay time constant tau2.
The normalized peak condunductance is 1.
Decay time MUST be greater than rise time.

The solution of A->G->bath with rate constants 1/tau1 and 1/tau2 is
 A = a*exp(-t/tau1) and
 G = a*tau2/(tau2-tau1)*(-exp(-t/tau1) + exp(-t/tau2))
      where tau1 < tau2

If tau2-tau1 -> 0 then we have a alphasynapse.
and if tau1 -> 0 then we have just single exponential decay.

The factor is evaluated in the
initial block such that an event of weight 1 generates a
peak conductance of 1.

Because the solution is a sum of exponentials, the
coupled equations can be solved as a pair of independent equations
by the more efficient cnexp method.

***
Modified to model an NMDA synapse
T Branco 2016
***

ENDCOMMENT

NEURON {
      POINT_PROCESS FDSExp2SynNMDA
:     USEION ca READ eca WRITE ica
      RANGE tau1, tau2, e, i
      NONSPECIFIC_CURRENT i
      RANGE g, caf
      GLOBAL total
        RANGE f, tau_F, d1, tau_D1, d2, tau_D2
}

UNITS {
      (nA) = (nanoamp)
      (mV) = (millivolt)
```

```
      (umho) = (micromho)
}

PARAMETER {
      tau1 = 0.1 (ms) < 1e-9, 1e9 >
      tau2 = 10 (ms) < 1e-9, 1e9 >
  mg=1    (mM)          : external magnesium concentration
  pf = 0.03  (1)       : adjusted to give 15% ica at -60 mV
      e = 0 (mV)
        : these values are from Fig.3 in Varela et al. 1997
      : the (1) is needed for the range limits to be effective
        f = 0.917 (1) < 0, 1e9 >    : facilitation
        tau_F = 94 (ms) < 1e-9, 1e9 >
        d1 = 0.416 (1) < 0, 1 >     : fast depression
        tau_D1 = 380 (ms) < 1e-9, 1e9 >
        d2 = 0.975 (1) < 0, 1 >     : slow depression
        tau_D2 = 9200 (ms) < 1e-9, 1e9 >
}


ASSIGNED {
      v (mV)
      i (nA)
      g (umho)
      factor
  eca (mV)
      ica (nA)
      total (umho)
}



STATE {
      A (umho)
      B (umho)
}

INITIAL {
      LOCAL tp
      total = 0
      if (tau1/tau2 > 0.9999) {
            tau1 = 0.9999*tau2
      }
      A = 0
      B = 0
      tp = (tau1*tau2)/(tau2 - tau1) * log(tau2/tau1)
      factor = -exp(-tp/tau1) + exp(-tp/tau2)
      factor = 1/factor
}

BREAKPOINT {
      SOLVE state METHOD cnexp
      g = B - A
  i = g*mgblock(v)*(v - e)*(1-pf)
:    ica = g*mgblock(v)*(v - eca)*pf
}

DERIVATIVE state {
```

```
        A' = -A/tau1
        B' = -B/tau2
}

FUNCTION mgblock(v(mV)) {
        TABLE
        DEPEND mg
        FROM -140 TO 80 WITH 1000

        : from Jahr & Stevens

        mgblock = 1 / (1 + exp(0.062 (/mV) * -v) * (mg / 3.57 (mM)))
}

NET_RECEIVE(weight (umho), F, D1, D2, tsyn (ms)) {
INITIAL {
: these are in NET_RECEIVE to be per-stream
          F = 1
          D1 = 1
          D2 = 1
          tsyn = t
: this header will appear once per stream
: printf("t\t t-tsyn\t F\t D1\t D2\t amp\t newF\t newD1\t newD2\n")
}

          F = 1 + (F-1)*exp(-(t - tsyn)/tau_F)
          D1 = 1 - (1-D1)*exp(-(t - tsyn)/tau_D1)
          D2 = 1 - (1-D2)*exp(-(t - tsyn)/tau_D2)
: printf("%g\t%g\t%g\t%g\t%g\t%g", t, t-tsyn, F, D1, D2, weight*F*D1*D2)
          tsyn = t

        state_discontinuity(A, A + weight*factor*F*D1*D2)
        state_discontinuity(B, B + weight*factor*F*D1*D2)
        total = total+weight*F*D1*D2

          F = F + f
          D1 = D1 * d1
          D2 = D2 * d2
: printf("\t%g\t%g\t%g\n", F, D1, D2)
}
```

```
import numpy as np
import neuron

from neuron import h
from neuron import load_mechanisms
from neuron import gui

load_mechanisms('./mod.files')
h('objref nil')

# -------------------------------------------------------------
# MODELS
class BS(object):
    def __init__(self, L=1.0, diam=1.0):       #dend L in lambda
        props(self)
        self._geom(L=L, diam=diam)
        self._topol()
        self._biophys()

    def _geom(self, L=1.0, diam=1.0):
        self.soma = h.Section()
        self.soma.nseg = 1
        self.soma.L=75
        self.soma.diam = self.soma.L

        self.dend = h.Section()
        self.dend.diam=diam
        self.dend.L =
L*np.sqrt(1e+4*(self.dend.diam/4.0)*(self.RM/self.RA))
        self.dend.nseg = 50
        print 'Dendritic length=', self.dend.L, ', with
nseg=',self.dend.nseg

        self.axon = h.Section()
        self.axon.L = 1#300
        self.axon.diam = 1

    def _topol(self):
        self.dend.connect(self.soma,1,0)
        self.axon.connect(self.soma,0,0)
        self.dends = []
        self.dends.append(self.dend)

    def _biophys(self):
        for sec in h.allsec():
            sec.cm = self.CM
            sec.insert('pas')
            sec.e_pas = self.E_PAS
            sec.g_pas = 1.0/self.RM
            sec.Ra = self.RA

# -------------------------------------------------------------
# INSTRUMENTATION FUNCTIONS
def props(model):

    # Passive properties
    model.CM = 1.0
    model.RM = 10000.0
```

```
    model.RA = 80.0
    model.E_PAS = -65
    model.CELSIUS = 35

    # Active properties
    model.Ek = -90
    model.Ena = 60
    model.Eca = 140

    model.gna_axon = 0000
    model.gkv_axon = 000

    model.gna_soma = 2000 * 0.0
    model.gkv_soma = 200 * 1
    model.gkm_soma = 2.2 * 1
    model.gkca_soma = 3 * 0
    model.gca_soma = 0.5 * 0
    model.git_soma = 0.0003 * 0

    model.gna_dend = 50
    model.gkv_dend = 10 * 0.8
    model.gkm_dend = 0.05 * 1
    model.gkca_dend = 3
    model.gca_dend = 0.5 * 1
    model.git_dend = 0.0003 * 0.5
    model.gh_dend = 0.00001 * 0

def taper(model):
    s = 0
    for seg in model.dend.allseg():
        #0.74 0.63
        if s<model.dend.nseg/2:
            seg.diam = 1
        else:
            seg.diam = 0.85
        s+=1

def init_active(model, axon=False, soma=False, dend=True, dendNa=False,
                dendCa=False):
    if axon:
        model.axon.insert('na'); model.axon.gbar_na = model.gna_axon
        model.axon.insert('kv'); model.axon.gbar_kv = model.gkv_axon
        model.axon.ena = model.Ena
        model.axon.ek = model.Ek

    if soma:
        model.soma.insert('na'); model.soma.gbar_na = model.gna_soma
        model.soma.insert('kv'); model.soma.gbar_kv = model.gkv_soma
        model.soma.insert('km'); model.soma.gbar_km = model.gkm_soma
        model.soma.insert('kca'); model.soma.gbar_kca = model.gkca_soma
        model.soma.insert('ca'); model.soma.gbar_ca = model.gca_soma
        model.soma.insert('it'); model.soma.gbar_it = model.git_soma
        model.soma.ena = model.Ena
        model.soma.ek = model.Ek
        model.soma.eca = model.Eca

    if dend:
        for d in model.dends:
```

```
            d.insert('na'); d.gbar_na = model.gna_dend*dendNa
            d.insert('kv'); d.gbar_kv = model.gkv_dend
            d.insert('km'); d.gbar_km = model.gkm_dend
            d.insert('kca'); d.gbar_kca = model.gkca_dend
            d.insert('ca'); d.gbar_ca = model.gca_dend*dendCa
            d.insert('it'); d.gbar_it = model.git_dend*dendCa
            d.ena = model.Ena
            d.ek = model.Ek
            d.eca = model.Eca


def add_AMPAsyns(model, locs=[[0, 0.5]], gmax=0.5, tau1=0.1, tau2=1):
    model.AMPAlist = []
    model.ncAMPAlist = []
    gmax = gmax/1000.   # Set in nS and convert to muS
    for loc in locs:
        AMPA = h.Exp2Syn(float(loc[1]), sec=model.dends[int(loc[0])])
        AMPA.tau1 = tau1
        AMPA.tau2 = tau2
        NC = h.NetCon(h.nil, AMPA, 0, 0, gmax)
        model.AMPAlist.append(AMPA)
        model.ncAMPAlist.append(NC)
    print len(locs), 'AMPA synapses added'

def add_fdsAMPAsyns(model, locs=[[0, 0.5]], gmax=0.5, f=0.9, tau_F=100,
                    d1=0.4, tau_D1=100, d2=1, tau_D2=9200, tau1=0.1,
tau2=1):
    model.AMPAlist = []
    model.ncAMPAlist = []
    gmax = gmax/1000.   # Set in nS and convert to muS
    for loc in locs:
        AMPA = h.FDSExp2Syn(float(loc[1]), sec=model.dends[int(loc[0])])
        AMPA.tau1 = tau1
        AMPA.tau2 = tau2
        AMPA.f = f
        AMPA.tau_F = tau_F
        AMPA.d1 = d1
        AMPA.tau_D1 = tau_D1
        AMPA.d2 = d2
        AMPA.tau_D2 = tau_D2
        NC = h.NetCon(h.nil, AMPA, 0, 0, gmax)
        model.AMPAlist.append(AMPA)
        model.ncAMPAlist.append(NC)
    print len(locs), 'fdsAMPA synapses added'

def add_NMDAsyns(model, locs=[[0, 0.5]], gmax=0.5, tau1=2, tau2=20):
    model.NMDAlist = []
    model.ncNMDAlist = []
    gmax = gmax/1000.   # Set in nS and convert to muS
    for loc in locs:
        NMDA = h.Exp2SynNMDA(float(loc[1]), sec=model.dends[int(loc[0])])
        NMDA.tau1 = tau1
        NMDA.tau2 = tau2
        NC = h.NetCon(h.nil, NMDA, 0, 0, gmax)
        x = float(loc[1])
        #NC = h.NetCon(h.nil, NMDA, 0, 0, gmax*(1+2*0.4*(x-1/2.))) #0.4
        model.NMDAlist.append(NMDA)
        model.ncNMDAlist.append(NC)
```

```
    print len(locs), 'NMDA synapses added'

def add_fdsNMDAsyns(model, locs=[[0, 0.5]], gmax=0.5, f=0.9, tau_F=100,
                    d1=0.4, tau_D1=100, d2=1, tau_D2=9200, tau1=2,
tau2=20):
    model.NMDAlist = []
    model.ncNMDAlist = []
    gmax = gmax/1000.   # Set in nS and convert to muS
    for loc in locs:
        NMDA = h.FDSExp2SynNMDA(float(loc[1]),
sec=model.dends[int(loc[0])])
        NMDA.tau1 = tau1
        NMDA.tau2 = tau2
        NMDA.f = f
        NMDA.tau_F = tau_F
        NMDA.d1 = d1
        NMDA.tau_D1 = tau_D1
        NMDA.d2 = d2
        NMDA.tau_D2 = tau_D2
        NC = h.NetCon(h.nil, NMDA, 0, 0, gmax)
        model.NMDAlist.append(NMDA)
        model.ncNMDAlist.append(NC)
    print len(locs), 'fdsNMDA synapses added'


# ------------------------------------------------------------
# SIMULATION RUN
def simulate(model, t_stop=100):
    # Set recording
    trec, vrec, grec = h.Vector(), h.Vector(), h.Vector()
    gRec, iRec, vDendRec = [], [], []
    trec.record(h._ref_t)
    vrec.record(model.soma(0.5)._ref_v)
    grec.record(model.AMPAlist[0]._ref_g)
    #grec.record(model.NMDAlist[0]._ref_g)
    # Run
    h.celsius = model.CELSIUS
    h.finitialize(model.E_PAS)
    neuron.run(t_stop)
    return np.array(trec), np.array(vrec), np.array(grec)
```

```
import numpy as np
import  time
import brian as br
from numpy.random import exponential, randint
from numpy import ones, cumsum, sum, isscalar

# Synapse location functions
def genAllLocs(isd=1):   # intersynaptic distance in microns
    locs = []
    dend_n = 0
    for dend in model.dends:
        distance = 0
        while distance<dend.L:
            locs.append([dend_n, distance/dend.L])
            distance = distance + isd
        dend_n = dend_n + 1
    return locs

def gen1dendLocs(dend, nsyn, spread):
    locs = []
    isd = (spread[1]-spread[0])/(nsyn)
    #isd = 1./(nsyn-1)
    pos = np.arange(spread[0], spread[1], isd)
    #pos = np.arange(0,1+isd,isd)
    for p in pos:
        #print p
        locs.append([dend, p])
    return locs

def genRandomLocs(nsyn):
    locs = []
    for s in np.arange(0,nsyn):
        dend = np.random.randint(low=0, high=len(model.dends))
        pos = np.random.uniform()
        locs.append([dend, pos])
    return locs

# Input generation functions
def genPoissonInput(nsyn, rate, duration, onset):
    times = np.array([])
    while times.shape[0]<2:
        P =  br.OfflinePoissonGroup(nsyn, rate, duration * br.ms)
        times = np.array(P.spiketimes)
    times[:,1] = times[:,1] * 1000 + onset
    rates = 1./np.diff(np.array(P.spiketimes)[:,1]).mean()
    return times#, rates
```

```
import numpy as np
import  h5py
import  time
import brian as br
import libcell as lb
import libinput as li


# Helper functions
def initOnsetSpikes(model, data):
    for n in range(len(model.ncAMPAlist)):
        model.ncAMPAlist[n].event(data['st_onset'])

def initSpikes(model, data):
    for s in data['etimes']:
        model.ncAMPAlist[int(s[0])].event(float(s[1]))
        if data['NMDA']: model.ncNMDAlist[int(s[0])].event(float(s[1]))

def storeSimOutput(data,v,g,r=None):
        data['vdata'].append(v)
        data['gdata'].append(g)
        if r is not None: data['rates'].append(r)

def dict2h5(d, h5parent):
    """ Converts a dictionary to hdf5.
    "d" is the dictionary
    "h5parent" is the root of the target .hdf5 file
    """
    for key in d.keys():
        if type(d[key]) is dict:
            group = h5parent.create_group(str(key))
            dict2h5(d[key], group)
        elif type(d[key]) is int:
            dset = h5parent.create_dataset(str(key), data=[d[key]])
        elif type(d[key]) is float:
            dset = h5parent.create_dataset(str(key), data=[d[key]])
        elif type(d[key]) is bool:
            dset = h5parent.create_dataset(str(key), data=[d[key]])
        elif type(d[key]) is list:
            c=0
            dgroup = h5parent.create_group(str(key))
            for i in d[key]:
                dset = dgroup.create_dataset(str(key)+'_'+str(c),
data=np.array(i).ravel())
                c+=1
        elif type(d[key]) is np.ndarray:
            try:
                if np.shape(d[key])[1]>0:
                    c=0
                    dgroup = h5parent.create_group(str(key))
                    for i in d[key]:
                        dset = dgroup.create_dataset(str(key)+'_'+str(c),
data=i.ravel())
                        c+=1
            except IndexError:
                dset = h5parent.create_dataset(str(key),
data=[d[key].ravel()])
        else:
```

```
            group = h5parent.create_group(str(key))


# Basic simulation functions
def SIM_poissonInput(model, data, rate, pr=1, save=False):
    """ Activate all synapses randomly at a fixed Poisson rate
    """
    duration = data['st_duration']
    data['etimes'] = li.genPoissonInput(data['Ensyn'], rate, duration,
                                        data['st_onset'])
    if pr<1:
        mask = np.random.rand(len(data['etimes']))<pr
        data['etimes'] = data['etimes'][mask]
    fih = lb.h.FInitializeHandler(1, (initSpikes,(model, data)))
    taxis, v, g = lb.simulate(model, t_stop=data['TSTOP'])
    storeSimOutput(data, v, g)
    data['taxis'] = taxis
    if save:
        np.save('./etimes', data['etimes'])

# Iterative simulation functions
def iSIM_poissonInput(model, data, rates, pr=1, save=False):
    """ Iterate rate in poisson train simulation
    rates - list or array with rates to run
    """
    etimes = []
    for t in range(data['TRIALS']):
        print 'Running trial', t, '......'
        for r in rates:
            SIM_poissonInput(model, data, r, pr)
            if save:
                etimes.append(data['etimes'])
        np.save('./etimes.npy', np.array(etimes))
```

```python
import numpy as np
import h5py
import matplotlib.pyplot as plt
import time
import brian as br
import libcell as lb
import libinput as li
import libsim as ls

# Create simulation parameter and data saving dictionary
data = {}

#-----------------------------------------------------------------------------
# Simulation CONTROL

# Timing
data['dt'] = 0.01  #0.01
data['st_onset'] = 200.0
data['st_duration'] = 500.
data['TSTOP'] = 1000
lb.h.dt = data['dt']
lb.h.steps_per_ms = 1.0/lb.h.dt

# Simulation
data['TRIALS'] = 50
data['simType'] = 'poissonInputIterate'
data['rateRange'] = np.arange(4,5,10)
data['iRange'] = np.arange(-0.1,0.2,0.1)
data['singleRate'] = 20
data['iterRates'] = np.arange(5,81,1)
data['tInterval'] = 0
data['nPulsesTrain'] = 20

# Model
data['model'] = 'BS'
data['locType'] = '1dend'
data['ACTIVE'] = True
data['ACTIVEdend'] = True
data['ACTIVEdendNa'] = True
data['ACTIVEdendCa'] = True
data['ACTIVEaxonSoma'] = False
data['SYN'] = True
data['SYNfds'] = True
data['SPINES'] = False
data['ICLAMP'] = False
data['NMDA'] = True
data['taper'] = False

# Synapses
data['loc1dend'] = 0
data['dendSpread'] = [0.5, 1.0]
data['Egmax'] = 0.5
data['NMDAgmax'] = 0.5
data['Ensyn'] = 15
data['facilitation'] = 2.5        #distal:2.5      proximal:1.6
data['fastDepression'] = 0.4      #distal:0.4      proximal:0.4
data['slowDepression'] = 0.94     #distal:0.94     proximal:0.94
data['pr'] = 1
```

```
# IClamp
data['iclampLoc'] = ['soma', 0.5]
data['iclampOnset'] = 50
data['iclampDur'] = 250
data['iclampAmp'] = 0
#----------------------------------------------------------------------------

# Create neuron and add mechanisms
if data['model'] == 'BS': model = lb.BS()
if data['model'] == 'Ball': model = lb.Ball()
if data['SPINES']: lb.addSpines(model)
if data['taper']: lb.taper(model)
if data['ACTIVE']: lb.init_active(model, axon=data['ACTIVEaxonSoma'],
                             soma=['data.ACTIVEaxonSoma'],
dend=data['ACTIVEdend'],
                             dendNa=data['ACTIVEdendNa'],
dendCa=data['ACTIVEdendCa'])

# Generate synapse locations
if data['locType']=='1dend':
    model.dends[data['loc1dend']].nseg = 50
    data['Elocs'] = li.gen1dendLocs(data['loc1dend'], data['Ensyn'],
data['dendSpread'])
if data['locType']=='random':
    data['Elocs'] = li.genRandomLocs(data['Ensyn'])

# Insert synapses
if data['SYN']:
    if data['SYNfds']:
        lb.add_fdsAMPAsyns(model, locs=data['Elocs'], gmax=data['Egmax'],
f=data['facilitation'],
                          d1=data['fastDepression'],
d2=data['slowDepression'])
    else:
        lb.add_AMPAsyns(model, locs=data['Elocs'], gmax=data['Egmax'])
    if data['NMDA']:
        if data['SYNfds']:
            lb.add_fdsNMDAsyns(model, locs=data['Elocs'],
gmax=data['NMDAgmax'], f=data['facilitation'],
                          d1=data['fastDepression'],
d2=data['slowDepression'])
        else:
            lb.add_NMDAsyns(model, locs=data['Elocs'],
gmax=data['Egmax'])


#----------------------------------------------------------------------------
# Data storage lists
data['vdata'], data['gdata'] = [], []
data['rates'] = []

#----------------------------------------------------------------------------
# Run simulation
if data['simType']=='poissonInputIterate':ls.iSIM_poissonInput(model,
data, data['iterRates'], data['pr'],save=True)

#----------------------------------------------------------------------------
```

```
# Save data
np.save('./data_distal_onlyTaper.npy', data['vdata'])
```
