# Supplementary material for: A Distance-Dependent Distribution of Presynaptic Boutons Tunes Frequency-Dependent Dendritic Integration
Source: Neuron. 2018 Jul 25;99(2):275–282.e3. doi: 10.1016/j.neuron.2018.06.015 (PMC6078905; doi:10.1016/j.neuron.2018.06.015)
Supplement: Document S2. Article plus Supplemental Information [file mmc3.pdf]

Report

Neuron

A Distance-Dependent Distribution of Presynaptic Boutons Tunes Frequency-Dependent Dendritic Integration

Highlights

- Presynaptic inputs decrease in size with distance along CA1 basal dendrites
- Release probability decreases with distance along basal dendrites
- Short-term facilitation increases with distance along basal dendrites
- Increased synaptic facilitation offsets passive decay and boosts supralinear events

Authors

Federico W. Grillo, Guilherme Neves, Alison Walker, Gema Vizcay-Barrena, Roland A. Fleck, Tiago Branco, Juan Burrone

Correspondence

juan.burrone@kcl.ac.uk

In Brief

Grillo et al. describe a structural and functional distance-dependent distribution of presynaptic properties along CA1 basal dendrites. Smaller distal synapses exploit greater synaptic short-term facilitation to overcome distance-related attenuation and trigger frequency-dependent active dendritic events.

Grillo et al., 2018, Neuron *99*, 275–282
July 25, 2018 © 2018 The Authors. Published by Elsevier Inc.
https://doi.org/10.1016/j.neuron.2018.06.015

CellPress

A Distance-Dependent Distribution of Presynaptic Boutons Tunes Frequency-Dependent Dendritic Integration

Federico W. Grillo,[1,2] Guilherme Neves,[1,2] Alison Walker,[1] Gema Vizcay-Barrena,[3] Roland A. Fleck,[3] Tiago Branco,[4] and Juan Burrone[1,2,5,*]
[1]Centre for Developmental Neurobiology, Kings College London, New Hunts House, Guys Hospital Campus, London, SE1 1UL, UK
[2]MRC Centre for Neurodevelopmental Disorders, Kings College London, New Hunts House, Guys Hospital Campus, London, SE1 1UL, UK
[3]Centre for Ultrastructural Imaging (CUI), Kings College London, New Hunts House, Guys Hospital Campus, London, SE1 1UL, UK
[4]The Sainsbury Wellcome Centre, University College London, 25 Howland Street, London, W1T 4JG, UK
[5]Lead Contact
*Correspondence: juan.burrone@kcl.ac.uk
https://doi.org/10.1016/j.neuron.2018.06.015

SUMMARY

How presynaptic inputs and neurotransmitter release dynamics are distributed along a dendritic tree is not well established. Here, we show that presynaptic boutons that form onto basal dendrites of CA1 pyramidal neurons display a decrease in active zone (AZ) size with distance from the soma, resulting in a distance-dependent increase in short-term facilitation. Our findings suggest that the spatial distribution of short-term facilitation serves to compensate for the electrotonic attenuation of subthreshold distal inputs during repeated stimulation and fine-tunes the preferred input frequency of dendritic domains.

INTRODUCTION

Pyramidal neurons receive thousands of excitatory inputs on their extensive dendritic arbors. As a consequence, neurons need strategies to balance the strength of their synaptic inputs so that signals arriving at distal synapses, far from the soma, have a meaningful contribution to neuronal output. Active dendritic integration can greatly boost synaptic signals by amplifying local depolarizations onto dendritic spikes that travel to the soma. Much has been done to describe this phenomenon, and its regulation, by focusing on postsynaptic compartments. In particular, previous studies have characterized the distribution of the size and strength of postsynaptic spines within dendritic domains and have established their contributions to dendritic integration (Branco and Häusser, 2010; Magee, 2000; Spruston, 2008). Overall, these findings showed that whereas apical dendrites show an increase in the strength of synaptic inputs with distance from the soma (Magee and Cook, 2000), the thinner basal and apical oblique dendrites show a decrease in spine size with distance (Katz et al., 2009; Menon et al., 2013; Walker et al., 2017). What emerges is a complex picture of synapse distribution along dendritic arbors that appear to favor either local (dendritic) or global (cell-wide) integration, depending on dendrite identity. On the other hand, we know much less about the structural and functional distribution of presynaptic boutons (de Jong et al., 2012) and the role they play in shaping the integration of synaptic inputs on dendrites and their subdomains (Chabrol et al., 2015). Critically, whereas postsynaptic strength establishes the amount of local dendritic depolarization, changes in presynaptic structure and function determine the dynamics of neurotransmitter release, through short-term forms of plasticity. This feature, in turn, governs the type of information that is transmitted across a synapse (Branco and Staras, 2009; Fioravante and Regehr, 2011). To understand how dendrites integrate synaptic inputs, it is therefore important to first uncover how presynaptic boutons and the dynamics of neurotransmitter release are distributed along dendrites. A non-random distribution that echoes that of postsynaptic spines could lead to specialized dendritic domains that code for specific streams of information. Thus, short-term forms of plasticity that govern the release of neurotransmitter across different presynaptic boutons may have profound effects on how information is processed in postsynaptic dendrites (Abrahamsson et al., 2012). More globally, short-term plasticity (STP) has been shown to play many important roles *in vivo* (Regehr, 2012) and, in excitatory synapses of the hippocampus, STP is thought to contribute to the transmission of information about an animal's place field (Kandaswamy et al., 2010; Klyachko and Stevens, 2006). Understanding the dynamics of neurotransmitter release in hippocampal presynaptic boutons will therefore also have implications for how information is encoded by CA1 neurons during active behaviors. Here, we show that presynaptic boutons decrease in size along the basal dendrites of CA1 hippocampal neurons, resulting in a decrease in release probability and an increase in short-term facilitation with distance from the soma. We reveal that this spatial distribution in STP tunes dendritic domains to specific information frequencies, introducing a further level of specialization to dendritic computations.

RESULTS

We set out to map the distribution of the structure and function of presynaptic terminals in CA1 *stratum oriens*, a region of the

This is an open access article under the CC BY license (http://creativecommons.org/licenses/by/4.0/).

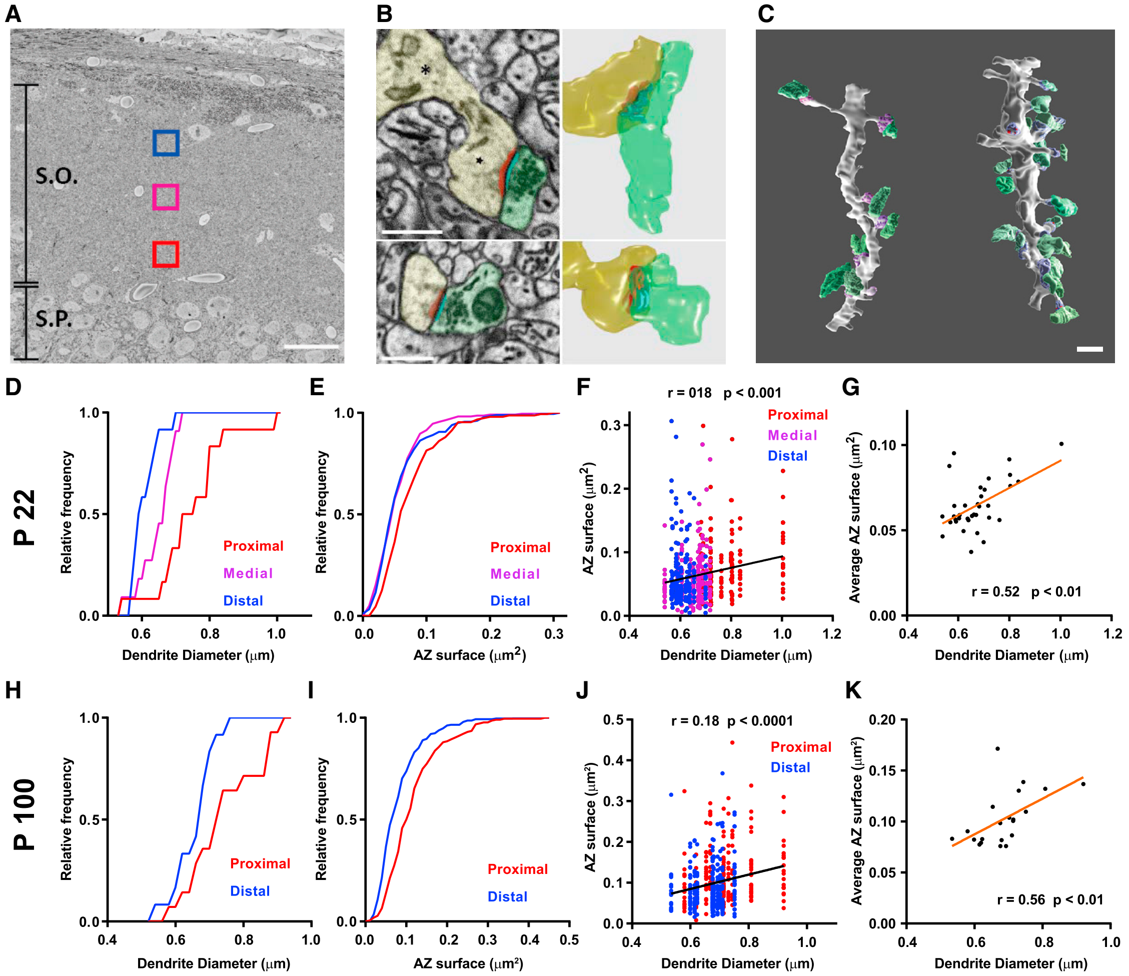

Figure 1. Presynaptic Active Zone Size Scales with Dendrite Diameter and Distance from Cell Bodies

(A) SBFSEM low-magnification image showing experimental design: 3 areas (red, proximal; purple, medial; blue, distal) at increasing distances from the pyramidal cell layer in the stratum oriens region of the CA1; S.O., stratum oriens; S.P., stratum pyramidale. Scale bar, 50 μm.

(B) SBFSEM single section images (left panels), with corresponding 3D reconstructions (right panels), showing presynaptic (yellow) and postsynaptic (green) structures with PSDs (red) and AZs (light blue) highlighted. Top left panel shows a spine head (star) connected through the narrower spine neck to the main dendritic shaft (asterisk). Scale bars, 0.5 μm.

(C) Two dendrites (thin dendrite left, thick dendrite right) reconstructed in 3D with spine heads in purple and boutons in green. Scale bar, 1 μm.

(D–G) Data from a postnatal day 22 animal. (D) Cumulative fraction plot: dendrites reconstructed in the proximal area have larger diameters than distal and medial area dendrites; n = 35 dendrites, $p < 0.001$ ANOVA; proximal-distal adjusted $p < 0.001$; proximal-medial adjusted $p < 0.01$, Tukey's multiple comparison test. (E) Cumulative fraction plot of AZ sizes, which are a larger in the proximal group; n = 604 AZs, $p < 0.0001$ Kruskal-Wallis test; proximal-medial adjusted $p < 0.0001$, proximal-distal adjusted $p = 0.0001$ Dunn's multiple comparison test. (F) Smaller AZs tend to be found on thinner dendritic processes, n = 556 AZs, Spearman's correlation; colors indicate area in which the dendrites were reconstructed. (G) Average AZ size (per dendrite) positively correlates with dendrite diameter, n = 35 dendrites, Spearman's correlation.

(H–K) Data from a postnatal day 100 animal. (H) Proximal dendrites are thicker than distal dendrites, cumulative fraction plot n = 26 dendrites, $p < 0.05$ unpaired t test with Welch's correction. (I) Cumulative fraction plot of AZ sizes, which are a larger in the proximal group; n = 505 AZs, $p < 0.0001$ Kolmogorov-Smirnov test. (J) Smaller AZs tend to be found on thinner dendritic processes, n = 505 AZs, Spearman's correlation; colors indicate area in which the dendrites were reconstructed. (K) Average AZ size (per dendrite) positively correlates with dendrite diameter, n = 26 dendrites, Spearman's correlation. See also Figure S1. Data are represented as mean ± SEM.

hippocampus that receives ordered axonal inputs that are mostly perpendicular to the basal dendrites of pyramidal neurons (Andersen et al., 1980). We first performed Serial Block-Face Scanning Electron Microscopy (SBFSEM) of three regions of CA1 *stratum oriens* from a P22 brain, ranging from deep (close to the *stratum pyramidale*) to superficial (close to *alveus*) areas and reconstructed basal dendrites together with their synaptic inputs (Figures 1A–1C). Although dendritic segments showed a wide distribution in the size of excitatory inputs, we found, as expected, a strong correlation between morphological measures of presynaptic and postsynaptic compartments (Figure S1) (Holderith et al., 2012; Schikorski and Stevens, 1997). In agreement

with the notion that basal dendrites taper toward tip ends (Menon et al., 2013), we also saw a decrease in dendrite diameter along the *stratum oriens* with increasing distance away from *stratum pyramidale* (Figure 1D; n = 35, mean = prox 0.75 ± 0.03 μm, med 0.65 ± 0.02 μm, dist 0.61 ± 0.01 μm, p < 0.001 Kruskal-Wallis test, prox-dist adjusted p < 0.001 Dunn's multiple comparison test). Importantly, of the many structural features measured at the synapse (Figure S1), AZ area, a good predictor of release probability (Holderith et al., 2012), showed a strong decrease in size with distance along *stratum oriens* (Figure 1E; n = 604, mean = prox 0.074 ± 0.003 μm^2, med 0.058 ± 0.002 μm^2, dist 0.061 ± 0.003 μm^2, p < 0.0001 Kruskal-Wallis test; prox-med adjusted p < 0.001, prox-dist adjusted p < 0.001 Dunn's multiple comparison test). Dendrite diameter, which can be taken as an indirect measure of distance from the soma along a tapering dendrite, also correlated well with AZ size (Figures 1F and 1G), providing further evidence that presynaptic terminals become smaller with distance along a basal dendrite. A reconstruction of proximal and distal dendrites performed on an adult brain (P100) showed a very similar distribution of presynaptic and postsynaptic properties with distance (Figures 1H–1K, Figure S1), indicating that the decrease in AZ size (and other synaptic parameters) was not unique to adolescent (P22) brains. Our dataset has some limitations, including the inability to follow the same dendrite from a single cell and to establish the identity of the inputs that arrive at different locations. We therefore turned to dissociated hippocampal neurons, where synaptic inputs can be labeled and followed along a single dendrite and where input identity is jumbled following the dissociation procedure. We find that measures of both presynaptic structure (vGlut labeling) and function (FM4-64 labeling of recycling vesicles) also decreased in a graded manner with distance along a dendrite (Figure S2). Together, these data suggest that the distance-dependent distribution of presynaptic inputs may be independent of the identity of the axon and is likely constrained by the properties of the postsynaptic dendrite.

AZ size is thought to correlate well with release probability (P_r) (Holderith et al., 2012; Murthy et al., 2001; Schikorski and Stevens, 1997). In addition, P_r is tightly coupled to the dynamics of neurotransmitter release during a burst of action potentials, such that high P_r synapses undergo short-term depression, whereas low P_r synapses exhibit short-term facilitation (Dobrunz and Stevens, 1997). This correlation between P_r and STP prompted us to measure neurotransmitter release dynamics along basal dendrites. We therefore patch clamped and filled CA1 pyramidal neurons with a fluorescent dye (Alexa 594) to visualize the basal dendritic arbor and measure synaptic inputs along its dendrites. Two stimulating pipettes were positioned along the basal dendrite, one at a site proximal to the soma and one distal to it (Figure 2A). In agreement with our structural findings (Figure 1), synaptic AMPA receptor currents measured by stimulating either proximal or distal axons independently showed that distal inputs facilitated more than proximal ones (Figures 2B and 2C). The paired-pulse ratio (PPR) of the first two stimuli delivered at 20 Hz was larger for distal inputs (mean = 1.59 ± 0.05) compared to proximal ones (mean = 1.33 ± 0.05) and remained higher throughout the 5 pulse stimulus train (Figures 2C and 2D). To establish whether the observed differences in STP were affected by postsynaptic properties, such as the inactivation or saturation state of AMPA receptors, we used cyclothiazide (CTZ, a drug that prevents AMPAR desensitization [Partin et al., 1993; Patneau et al., 1993]) or γDGG (a competitive antagonist of glutamate receptors [Liu et al., 1999]) to directly assess any contributions to STP by postsynaptic receptors (Figure 2E). Neither drug had any effect on the difference in STP between distal and proximal inputs (CTZ PPR = prox 1.18 ± 0.09, dist 1.51 ± 0.09, n = 9, p = 0.01; γDGG PPR = prox 1.36 ± 0.07, dist 1.64 ± 0.09, n = 13, p < 0.01; two-way ANOVA for control, CTZ, and γDGG, interaction p = 0.84), although there was a positive trend of γDGG on facilitation (Figure S3) that could reflect multivesicular release during the train (Christie and Jahr, 2006; Oertner et al., 2002; Wadiche and Jahr, 2001). To further corroborate that these effects were indeed presynaptic in origin, we performed similar measures of PPR in transgenic mice where synaptotagmin 7 (Syt 7) was knocked out. Syt 7$^{-/-}$ mice have been shown to lack any facilitation (Jackman et al., 2016) and should therefore abolish the differences in PPR measured here. We find that whereas WT mice show the same distance-dependent increase in PPR observed above, Syt7$^{-/-}$ littermates show neither facilitation nor any obvious difference in PPR between proximal and distal domains (Figure 2F). Together, our data point to a presynaptic origin in the difference in PPR along basal dendrites.

To confirm the location specificity of our stimulus pipettes, we measured the kinetics of synaptic transmission at the soma. In agreement with the electrotonic decay of signals along a dendrite (Mainen et al., 1996; Rall, 1962; Spruston et al., 1994), we confirmed that distal stimuli (mean = 1.66 ± 0.07 ms) elicited slower events than proximal stimuli (mean = 1.23 ± 0.09 ms; p < 0.001, Wilcoxon sign rank test; Figure 2G). Since both the rise time and time to peak of events are directly related to the distance from the recording pipette at the soma, we looked at their relationship with PPR across different cells. A positive correlation emerged between synaptic kinetics and PPR (Figure 2H and Figures S3F and S3G, n = 49), suggesting a gradual change in PPR along a dendrite. Indeed, a positive correlation was also observed when PPR was plotted against the absolute distance of the stimulating electrode from the soma (Figure 2I). Our structural observations (Figure 1) predict that distal synapses will have a lower release probability (P_r) than proximal ones (Holderith et al., 2012), which may, in turn, help account for the increased levels of distal facilitation. To test for this, we used the irreversible open-channel blocker of NMDA receptors (NMDARs), MK-801, to measure P_r at proximal and distal compartments (Figure 2J). We recorded NMDAR currents in the presence of MK-801 in response to successive stimuli to measure the gradual block of NMDAR channels. The decay was best fit by a double exponential function, with a fast and a slow phase, indicating at least two groups of synapses with high and low release probabilities, respectively (Hessler et al., 1993). The two time constants were very similar for proximal and distal dendrites (proximal, τ1: 1.44 and τ2: 14.64; distal, τ1: 1.37 and τ2: 16.14), but in distal dendrites a larger fraction of the decay was explained by the slower time constant (proximal: 49%; distal: 69%). The overall slower decay observed in distal dendrites (mean half-life, proximal: 7.28 ± 1.2, distal: 13.99 ± 3.03, n = 8, p = 0.027, paired t test)

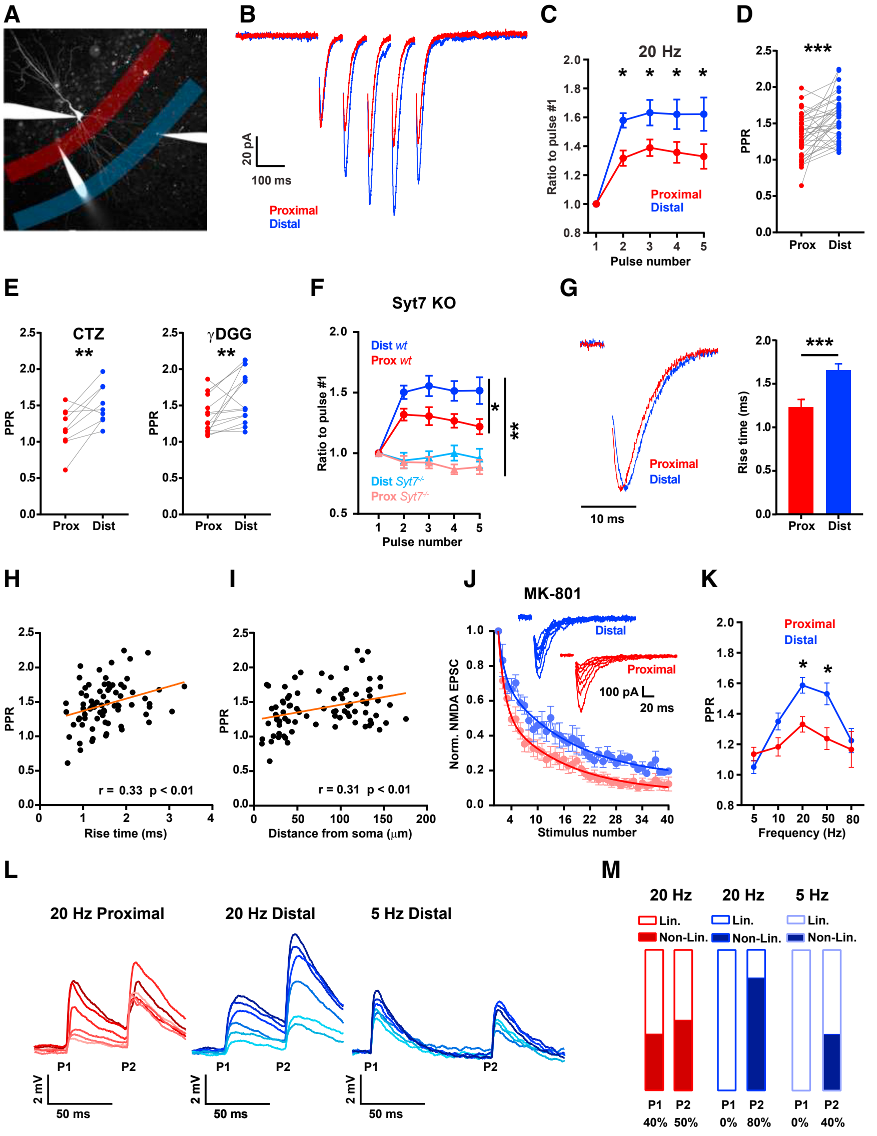

Figure 2. Increases in Short-Term Facilitation with Distance along a Dendrite Boost Distal Synaptic Integration

(A) Whole-cell patch-clamp technique was used to record synaptic currents and fill CA1 pyramidal cells with a fluorescent dye to image its structure. Two stimulating pipettes were placed in the proximal (red) and distal (blue) extracellular domains of the basal dendritic tree of pyramidal cells to stimulate the local fibers.

(B) Single cell example of average EPSC responses (average of 20 individual sweeps) to trains of 5 pulses at 20 Hz delivered to the proximal region (red trace) and the distal region (blue trace). The distal response shows greater facilitation compared to the proximal one.

(C) Normalized average peak EPSC amplitudes for distal and proximal responses show greater sustained facilitation during a 5 pulse train (20 Hz) for distal synapses, n = 35 cells, multiple t tests with p values adjusted with the Holm-Sidak method, $p < 0.05$.

(D) Paired-pulse ratios (PPRs) for each individual cell recorded at distal and proximal synapses. The majority (29/35) of cells display greater facilitation in the distal domain, n = 35 cells, p = 0.0003 two-tailed paired t test.

(E) Distal increase in PPR is not ascribable to postsynaptic AMPA receptor desensitization (prevented by CTZ application) or to AMPA receptor saturation (avoided with γDGG application). Distal PPR is greater than proximal PPR with CTZ (n = 9 cells, p = 0.01), and γDGG (n = 13 cells, $p < 0.01$), two-tailed paired t test. Two-way ANOVA to test PPRs in control (D), CTZ, and γDGG conditions together shows no significant interaction, p = 0.84, indicating that the drugs have no effect on STP properties.

(F) Full Synaptotagmin7 KO eliminates facilitation and proximo-distal STP differences. Triangles in lighter colors are from Syt7KO mice, n = 9 cells, circles in darker colors are littermate wild-type mice, n = 12 cells. For WT mice, proximal facilitation is lower than distal, multiple t tests, $p < 0.05$. WT facilitation is greater than Syt7KO facilitation, $p < 0.01$ multiple t tests.

(G–I) Distal synaptic EPSCs take longer to reach the soma. (G) Left panel: normalized trace for a proximal and distal EPSC response showing the delayed kinetics of the distal compared to the proximal synaptic current. Right panel: the rise time constant of the EPSCs was significantly higher in distally triggered events, n = 49 cells, $p < 0.001$ Wilcoxon signed rank test. (H) Longer rise times correlate with the amount of facilitation, Spearman's correlation. (I) PPR is higher when the stimulation electrode is placed further away from the soma, measured as distance along the dendrite, Spearman's correlation.

(J) Proximal synapses display greater P_r than distal synapses. After MK-801 bath application, the normalized amplitude of EPSCs from proximally stimulated synapses decay faster, following successive stimulations, than distal ones; n = 8 cells. Data points were fit with a double exponential function (filled lines). Insets are example traces of 7 successive NMDA mediated EPSCs.

(K) Frequency tuning curve showing PPRs for all frequencies tested. Distal PPRs (second stimulus only) increase significantly in the 20 Hz (n = 35) same as (D), and 50 Hz range (n = 20), $p < 0.05$, multiple t tests with Holm-Sidak adjusted p values. 5 Hz (n = 13 cells), 10 Hz (n = 16), 80 Hz (n = 9).

(L–M) Short-term facilitation contributes to dendritic non-linear events in distal domains. (L) Current-clamp example traces (red proximal, blue distal stimulation) in response to a paired pulse, of increasing stimulus intensity (lighter color shades represent lower intensity). (M) The proportion of supra-linear events (at least 2 mV above the expected response) for distal synapses is greatly increased for the second pulse (P2) following facilitation at 20 Hz (0 events in P1, 8 events in P2, n = 10), while supra-linear events were detected to the first pulse (P1) for proximal stimulations (n = 10, 4 in P1, 5 in P2). At 5 Hz, where STP is absent, distal synapses had fewer supra-linear events (n = 5, 0 in P1, 2 in P2). See also Figures S2 and S3. Data are represented as mean ± SEM.

indicates that synaptic NMDARs experienced less neurotransmitter in response to successive stimuli and therefore belong to synapses with, on average, a lower P_r than those found in proximal dendrites (Figure 2J). These findings mirror the structural correlation of presynaptic AZ size with dendrite diameter (Figures 1G and 1K) and strengthen our hypothesis that both

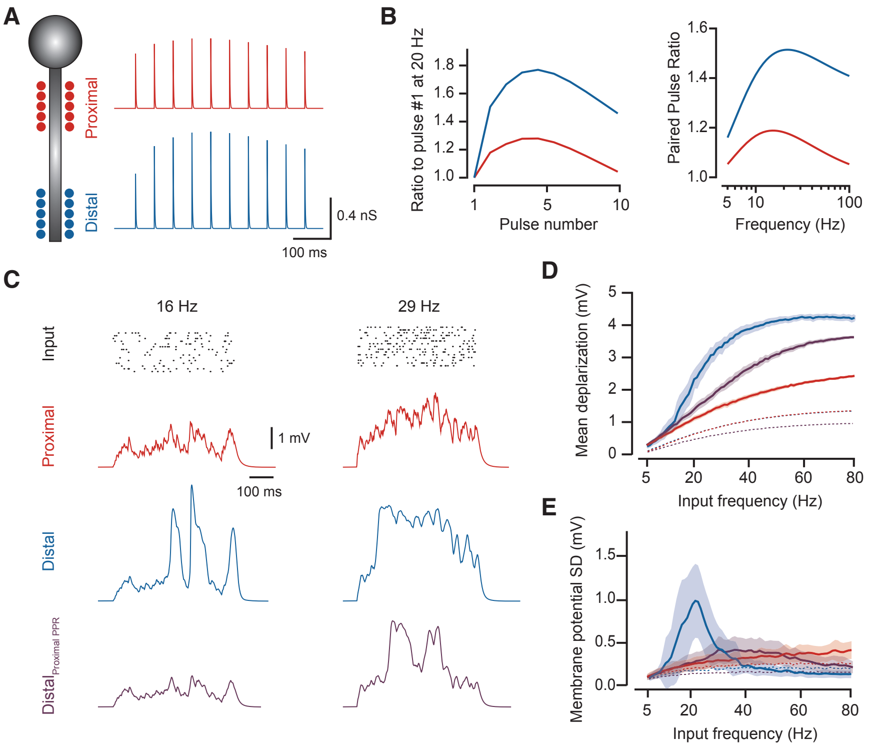

Figure 3. PPR Gradient Enhances Distal Supra-linear Integration

(A) Compartmental model illustration with distal and proximal synapses along one dendritic branch (left) and respective single synapse AMPA conductance traces for 20 Hz stimulation (right).

(B) Model data showing PPR dynamics for each pulse in a train of 20 Hz (left) and for PPR between 5 and 100 Hz for the first two pulses in the train.

(C) Example simulation traces for Poisson input trains delivered independently at each synapse at two different rates. Top raster shows input times for all synapses, and traces below show responses to the same input for proximal (red) and distal (blue) synapses, and for distal synapses equipped with PPR identical to proximal synapses (purple).

(D and E) (D) Mean depolarization during stimulation for different input frequencies and (E) respective standard deviation of the membrane potential, showing that increased PPF at distal synapses enhances supra-linear integration. Dashed lines in (D) and (E) are for passive models for each position (red proximal, blue distal, and purple distal with proximal PPR properties); note that in (D) the red and blue dashed lines overlap. See also Figure S4. Data are represented as mean ± SEM.

the structure and function of presynaptic boutons display a distance-dependent distribution along basal dendrites. Furthermore, the lack of cross-inhibition by MK-801 between proximal and distal inputs is further support for the specificity of our stimulating electrodes in recruiting axons that form connections locally (Figure S3). Finally, we also established the frequency range over which inputs showed facilitation. We found that distal inputs showed significantly larger facilitation than proximal ones over a limited frequency range, between 10 and 50 Hz (Figures 2K and S3), with a peak at 20 Hz (PPR means = 5 Hz, prox 1.13 ± 0.04, dist 1.05 ± 0.04; 10 Hz, prox 1.18 ± 0.06, dist 1.35 ± 0.05; 20 Hz, see above; 50 Hz, prox 1.24 ± 0.07, $p < 0.01$ dist 1.53 ± 0.07, $p < 0.01$; 80 Hz, prox 1.17 ± 0.12, dist 1.22 ± 0.08). So far, our data show that distal inputs are tuned to band-pass frequencies in the near gamma frequency range, which curiously match the high-frequency discharges measured *in vivo* in hippocampal place cells when an animal passes through a place field (Huxter et al., 2003).

To establish whether the biased distribution in short-term facilitation can influence dendritic integration and neuronal output, we performed current-clamp experiments and locally stimulated either proximal or distal afferents, as above. We found that stimulation of distal inputs at frequencies that showed increased facilitation (20 Hz), also showed a tendency to produce non-linear summations to multiple stimuli across the range of stimulation intensities (Figures 2L and 2M). This effect was less pronounced in proximal dendrites, where non-linear events were only observed for high stimulus intensities. The difference in integration properties between proximal and distal events has been shown to depend on the opening of NMDARs (Ariav et al., 2003; Branco and Häusser, 2011; Major et al., 2008; Schiller et al., 2000), which are more likely to be activated at the high impedance distal dendrites, where levels of depolarization to a given input are higher. We found that distal inputs are better suited to respond to multiple stimuli than to a single stimulus. This was apparent when comparing responses along a paired pulse. The second stimulus of a 20 Hz pair was consistently more likely to summate in a supralinear manner, when compared either to the first stimulus or to stimuli delivered at 5 Hz (Figures 2L and 2M). Together, our data show that the integration properties of distal domains are markedly different from proximal ones and depend on input frequency. Although there are many factors that likely play a role in driving these differences in dendritic integration, STP is an obvious candidate. To explore this further, we turned to a computational model where we could directly assess the role played by STP on the integration properties of different dendritic domains.

We built a model consisting of a cluster of proximal and distal synapses (15 each; Figure 3A), where STP was adjusted to match our data (Figure 3B). This simple model aims to first determine the impact that a distance-dependent STP gradient can have on dendritic integration, when all other variables are the same. Activating either proximal or distal synapses separately with Poisson input trains delivered at different mean frequencies elicited non-linear responses (Figure 3C). Distal inputs, however, showed larger levels of membrane depolarization compared to proximal ones, over a large frequency range (continuous lines in Figure 3D). This distal amplification of synaptic inputs was partially lost when distal synapses were switched to proximal STP properties, suggesting that increased levels of facilitation

enhance supralinear integration in distal dendrites. Interestingly, the difference in STP along a dendrite not only played a role in modulating dendritic non-linearities, it also contributed to distance-dependent input normalization; removal of active conductances from the model showed similar levels of membrane depolarization for distal and proximal inputs, which was lost when distal synapses were tuned to proximal STP properties (dashed lines in Figure 3D; note that the red and blue dashed lines overlap). This result suggests that the gradient in STP can boost distal inputs to counteract the effects of passive decay. Finally, increased levels of facilitation also resulted in an increased variance of membrane responses over a limited frequency domain, given by the short-term dynamics of the synapse (Figure 3E). This model shows that the STP gradient can have a significant impact on synaptic input integration, mainly exploiting differences in the recruitment of dendritic non-linearities. As such, it will act alongside other mechanisms that impact on non-linear integration, such as synaptic conductances, impedance, or release probability, all of which can also display gradients. However, models that include additional gradients of these other variables showed that the input-output curve remains biased toward larger depolarizations at higher frequencies in distal dendrites (Figure S4), indicating that the magnitude of the STP gradient that we observe is sufficient to have a significant impact on dendrite integration across a variety of conditions. Together, these simulations support the idea that distal inputs have an increased capacity for information transfer that is boosted by the short-term dynamics of neurotransmitter release from presynaptic inputs.

DISCUSSION

Our findings uncovered a gradient in the distribution of presynaptic terminals along dendrites that dictates the short-term dynamics of synaptic transmission. This spatial gradient in short-term facilitation serves to both normalize the distance-dependent decay in the amplitude of subthreshold inputs to repeated stimuli, as well as tune the preferred input frequency of different dendritic domains through supralinear integration. As a result, the type of information transmitted across a synapse will depend on its location along a dendrite, an arrangement that could be exploited by different input streams to achieve input-specific differential integration on the same dendrite.

Previous studies have shown that pre- and post-synaptic compartments are well matched, both structurally and functionally (Kay et al., 2011; Murthy et al., 2001). Our findings of a spatial bias in the distribution of presynaptic boutons would predict that postsynaptic structures should follow suit. Indeed, work focusing on postsynaptic spines has shown that along the basal dendrites of CA1 pyramidal neurons, non-perforated spines, which represent the great majority of excitatory inputs, show a decrease in size (including PSD size) with dendritic distance (Menon et al., 2013; Walker et al., 2017). This reduction in size is thought to locally compensate for the increased impedance of thinner, distal dendrites, normalizing responses locally, rather than cell-wide (Katz et al., 2009). By including presynaptic boutons and neurotransmitter release dynamics, our findings uncover spatially segregated domains for the transfer of information within dendrites. However, our experiments provide no information on the identity of the axons that innervate different dendritic compartments along the *stratum oriens*. In general, it is thought that axons in this region of the hippocampus arrive mainly from pyramidal cells in areas CA3 and CA2, although other brain areas may be involved. There is, in fact, a biased topographic projection of CA3 pyramidal cells to the *stratum oriens*, such that CA3 cells whose soma lie closer to the dentate gyrus (DG) project preferentially to distal dendritic domains of CA1 neurons, whereas those CA3 cells further away from the DG project to more proximal dendritic domains (Ishizuka et al., 1990). It is therefore possible that the properties of distal and proximal boutons are dictated by the identity of the presynaptic neuron itself. However, our *in vitro* findings argue against this view. Using dissociated hippocampal neurons, we show that a similar distance-dependent decrease in presynaptic bouton properties also occurs *in vitro* (Figure S2) (de Jong et al., 2012) and suggests that the distribution of synapses observed *in vivo* may well be independent of the circuit or the identity of the synaptic input. It remains a likely possibility that the biased synaptic distribution along dendrites is therefore specified by the postsynaptic neuron in a cell-autonomous manner. Indeed, work in dissociated hippocampal neurons has shown that local dendritic depolarization is a major determinant of presynaptic release probability (P_r), such that increases in depolarization induce a homeostatic decrease in P_r (Branco et al., 2008). It is therefore tempting to speculate that the increase in impedance in distal dendrites, which would result in larger synaptic amplitudes (Spruston, 2008), would in turn act to reduce P_r locally. In this way, dendritic impedance could act as a readout of dendritic distance. Possible molecular mechanisms may include retrograde messengers, some of which have been shown to modulate presynaptic function in response to postsynaptic membrane depolarization (Regehr et al., 2009).

Our data show that synapses are distributed in a distance-dependent manner along dendrites and we provide multiple lines of evidence to suggest that this distribution is also graded with distance. First, we find a graded correlation between synapse morphology and dendrite diameter (Figures 1G and 1K), a measure that has been previously shown to be a good proxy for distance along a given dendrite (Walker et al., 2017). Second, measures of PPR are correlated to the time course of the EPSC (Figure 2H), reflecting the distance-dependent filtering suffered by an EPSC as it travels to the soma. However, these measures of distance are indirect and incur a certain amount of noise that likely arises from the fact that dendrites are heterogenous entities, especially across different cells, with variable degrees of tapering and where the passive electrotonic decay of synaptic events can be influenced by other factors. Importantly, direct measures of dendritic distance in either acute slices (Figure 2I) or in primary neuronal cells (Figure S2) both show a correlation with presynaptic properties. Together, our data support the notion of a graded distribution of synaptic structure and function along dendrites.

Our study finds a strong correlation between presynaptic structure and function, where boutons with smaller AZs have a

lower P_r and an increased PPR. However, although PPR has been shown to correlate with the overall P_r of a bouton (Dobrunz and Stevens, 1997), this correlation may well be driven by the release probability of individual vesicles (P_{vr}) along an active zone. Differences in P_{vr} can arise from a number of different scenarios, ranging from structural features (e.g., the relative distribution of vesicles and calcium channels) to molecular heterogeneity (e.g., the expression of specific proteins that influence exocytosis). Although we have not explored these possibilities in our study, they could also underlie some of the differences in PPR observed here.

One other recent study has also shown a graded distribution of short-term plasticity along dendrites (Abrahamsson et al., 2012), although both the mechanism and direction of the gradient were opposed to that shown here. Stellate cells, a type of interneuron in the cerebellum, show a decrease in short-term facilitation along their thin dendrites (Abrahamsson et al., 2012). However, the effect is purely postsynaptic and arises from the large local depolarization in distal dendritic domains that reduces the driving force of postsynaptic receptors, resulting in the sub-linear integration of clustered inputs. As a result, stellate cells become ideal integrators of decorrelated inputs in both space and time, preferring the arrival of sparsely distributed, asynchronous events. This is clearly distinct form the mechanisms preferentially used by basal dendrites in the hippocampus and cortex, where clustered inputs drive local supralinear dendritic events (Branco and Häusser, 2011; Losonczy and Magee, 2006; Makara and Magee, 2013). Indeed, our data show that in CA1 basal dendrites, facilitation is distributed in the opposite direction to stellate cells, increasing with distance from the soma. Furthermore, according to our experimental data and computational model, increased distal facilitation is needed to trigger supralinear dendritic integration as this requires fast concomitant activation of multiple clustered synapses (Branco and Häusser, 2011), the likelihood of which is boosted by presynaptic facilitation. Collectively, these studies underscore the importance of short-term forms of plasticity on dendritic integration.

Finally, the difference in STP in proximal versus distal dendrites will also have important consequences on the way each domain encodes information. Proximal synapses that facilitate less will be better suited to respond to more isolated or lower-frequency inputs, suggesting that they respond better to temporally decorrelated events. Distal dendrites, on the other hand, with their higher levels of facilitation will act as spatio-temporal filters that favor high-frequency, clustered inputs. These highly selective distal inputs may, therefore, carry salient information for working memory or place field location. Facilitation of synaptic transmission in excitatory hippocampal synapses has been proposed to act as an adaptive high-pass filter that transmits and amplifies the signals encoding place-field information (Kandaswamy et al., 2010; Klyachko and Stevens, 2006). Our data suggest that the distal domains of CA1 basal dendrites are better suited to perform this computation. Future work will need to establish the identity of the inputs that arrive along different dendritic locations and understand the type of information they encode.

STAR★METHODS

Detailed methods are provided in the online version of this paper and include the following:

- KEY RESOURCES TABLE
- CONTACT FOR REAGENT AND RESOURCE SHARING
- METHOD DETAILS
    - Animals
    - Dissociated hippocampal cultures
    - Electron Microscopy
    - Electrophysiology
    - Modeling
    - Immuno-histochemistry and FM4-64 staining of primary neurons
    - Statistics

SUPPLEMENTAL INFORMATION

Supplemental Information includes four figures and supplemental data and can be found with this article online at https://doi.org/10.1016/j.neuron.2018.06.015.

ACKNOWLEDGMENTS

We would like to thank Alejandro Pan Vazquez, David Expósito Alonso and Erin Cocks for help with the EM image segmentation and reconstruction, Thomas Sainsbury for help with electrophysiology experiments. We would also like to thank Matthew Grubb, Oscar Marin, and members of the Burrone lab for critical feedback of the manuscript. This work was supported by an ERC Starter Grant (282047), a Wellcome Trust Investigator Award (095589/Z/11/Z), and an FP7 EC DESIRE grant (602531) to J.B.; a Wellcome Trust/Royal Society Henry Dale Fellowship (098400/Z/12/Z) and a Wellcome Trust and Gatsby Charitable Foundation SWC Fellowship to T.B., and a Wellcome Trust Multiuser Equipment grant (108461/Z/15/Z) to R.A.F.

AUTHOR CONTRIBUTIONS

F.W.G. and J.B. designed the study and wrote the text. F.W.G. performed and analyzed the experiments with help from G.N. who also gave critical input. G.V.-B. prepared the EM samples and acquired EM images, R.A.F. provided EM support. A.W. performed and analyzed the experiments on dissociated neurons. T.B. produced the computational model.

DECLARATION OF INTERESTS

The authors declare no competing interests.

Received: March 24, 2017
Revised: March 23, 2018
Accepted: June 8, 2018
Published: July 5, 2018

REFERENCES

Abrahamsson, T., Cathala, L., Matsui, K., Shigemoto, R., and Digregorio, D.A. (2012). Thin dendrites of cerebellar interneurons confer sublinear synaptic integration and a gradient of short-term plasticity. Neuron *73*, 1159–1172.

Andersen, P., Silfvenius, H., Sundberg, S.H., and Sveen, O. (1980). A comparison of distal and proximal dendritic synapses on CAi pyramids in guinea-pig hippocampal slices in vitro. J. Physiol. *307*, 273–299.

Ariav, G., Polsky, A., and Schiller, J. (2003). Submillisecond precision of the input-output transformation function mediated by fast sodium dendritic spikes in basal dendrites of CA1 pyramidal neurons. J. Neurosci. *23*, 7750–7758.

Branco, T., and Häusser, M. (2010). The single dendritic branch as a fundamental functional unit in the nervous system. Curr. Opin. Neurobiol. *20*, 494–502.

Branco, T., and Häusser, M. (2011). Synaptic integration gradients in single cortical pyramidal cell dendrites. Neuron *69*, 885–892.

Branco, T., and Staras, K. (2009). The probability of neurotransmitter release: variability and feedback control at single synapses. Nat. Rev. Neurosci. *10*, 373–383.

Branco, T., Staras, K., Darcy, K.J., and Goda, Y. (2008). Local dendritic activity sets release probability at hippocampal synapses. Neuron *59*, 475–485.

Cardona, A., Saalfeld, S., Schindelin, J., Arganda-Carreras, I., Preibisch, S., Longair, M., Tomancak, P., Hartenstein, V., and Douglas, R.J. (2012). TrakEM2 software for neural circuit reconstruction. PLoS ONE *7*, e38011.

Chabrol, F.P., Arenz, A., Wiechert, M.T., Margrie, T.W., and DiGregorio, D.A. (2015). Synaptic diversity enables temporal coding of coincident multisensory inputs in single neurons. Nat. Neurosci. *18*, 718–727.

Chakrabarti, S., Kobayashi, K.S., Flavell, R.A., Marks, C.B., Miyake, K., Liston, D.R., Fowler, K.T., Gorelick, F.S., and Andrews, N.W. (2003). Impaired membrane resealing and autoimmune myositis in synaptotagmin VII-deficient mice. J. Cell Biol. *162*, 543–549.

Christie, J.M., and Jahr, C.E. (2006). Multivesicular release at Schaffer collateral-CA1 hippocampal synapses. J. Neurosci. *26*, 210–216.

de Jong, A.P., Schmitz, S.K., Toonen, R.F., and Verhage, M. (2012). Dendritic position is a major determinant of presynaptic strength. J. Cell Biol. *197*, 327–337.

Dobrunz, L.E., and Stevens, C.F. (1997). Heterogeneity of release probability, facilitation, and depletion at central synapses. Neuron *18*, 995–1008.

Fioravante, D., and Regehr, W.G. (2011). Short-term forms of presynaptic plasticity. Curr. Opin. Neurobiol. *21*, 269–274.

Hessler, N.A., Shirke, A.M., and Malinow, R. (1993). The probability of transmitter release at a mammalian central synapse. Nature *366*, 569–572.

Hines, M.L., and Carnevale, N.T. (1997). The NEURON simulation environment. Neural Comput. *9*, 1179–1209.

Holderith, N., Lorincz, A., Katona, G., Rózsa, B., Kulik, A., Watanabe, M., and Nusser, Z. (2012). Release probability of hippocampal glutamatergic terminals scales with the size of the active zone. Nat. Neurosci. *15*, 988–997.

Huxter, J., Burgess, N., and O'Keefe, J. (2003). Independent rate and temporal coding in hippocampal pyramidal cells. Nature *425*, 828–832.

Ishizuka, N., Weber, J., and Amaral, D.G. (1990). Organization of intrahippocampal projections originating from CA3 pyramidal cells in the rat. J. Comp. Neurol. *295*, 580–623.

Jackman, S.L., Turecek, J., Belinsky, J.E., and Regehr, W.G. (2016). The calcium sensor synaptotagmin 7 is required for synaptic facilitation. Nature *529*, 88–91.

Jorstad, A., Nigro, B., Cali, C., Wawrzyniak, M., Fua, P., and Knott, G. (2015). NeuroMorph: a toolset for the morphometric analysis and visualization of 3D models derived from electron microscopy image stacks. Neuroinformatics *13*, 83–92.

Kandaswamy, U., Deng, P.Y., Stevens, C.F., and Klyachko, V.A. (2010). The role of presynaptic dynamics in processing of natural spike trains in hippocampal synapses. J. Neurosci. *30*, 15904–15914.

Katz, Y., Menon, V., Nicholson, D.A., Geinisman, Y., Kath, W.L., and Spruston, N. (2009). Synapse distribution suggests a two-stage model of dendritic integration in CA1 pyramidal neurons. Neuron *63*, 171–177.

Kay, L., Humphreys, L., Eickholt, B.J., and Burrone, J. (2011). Neuronal activity drives matching of pre- and postsynaptic function during synapse maturation. Nat. Neurosci. *14*, 688–690.

Klyachko, V.A., and Stevens, C.F. (2006). Excitatory and feed-forward inhibitory hippocampal synapses work synergistically as an adaptive filter of natural spike trains. PLoS Biol. *4*, e207.

Liu, G., Choi, S., and Tsien, R.W. (1999). Variability of neurotransmitter concentration and nonsaturation of postsynaptic AMPA receptors at synapses in hippocampal cultures and slices. Neuron *22*, 395–409.

Losonczy, A., and Magee, J.C. (2006). Integrative properties of radial oblique dendrites in hippocampal CA1 pyramidal neurons. Neuron *50*, 291–307.

Magee, J.C. (2000). Dendritic integration of excitatory synaptic input. Nat. Rev. Neurosci. *1*, 181–190.

Magee, J.C., and Cook, E.P. (2000). Somatic EPSP amplitude is independent of synapse location in hippocampal pyramidal neurons. Nat. Neurosci. *3*, 895–903.

Mainen, Z.F., Carnevale, N.T., Zador, A.M., Claiborne, B.J., and Brown, T.H. (1996). Electrotonic architecture of hippocampal CA1 pyramidal neurons based on three-dimensional reconstructions. J. Neurophysiol. *76*, 1904–1923.

Major, G., Polsky, A., Denk, W., Schiller, J., and Tank, D.W. (2008). Spatiotemporally graded NMDA spike/plateau potentials in basal dendrites of neocortical pyramidal neurons. J. Neurophysiol. *99*, 2584–2601.

Makara, J.K., and Magee, J.C. (2013). Variable dendritic integration in hippocampal CA3 pyramidal neurons. Neuron *80*, 1438–1450.

Menon, V., Musial, T.F., Liu, A., Katz, Y., Kath, W.L., Spruston, N., and Nicholson, D.A. (2013). Balanced synaptic impact via distance-dependent synapse distribution and complementary expression of AMPARs and NMDARs in hippocampal dendrites. Neuron *80*, 1451–1463.

Murthy, V.N., Schikorski, T., Stevens, C.F., and Zhu, Y. (2001). Inactivity produces increases in neurotransmitter release and synapse size. Neuron *32*, 673–682.

Oertner, T.G., Sabatini, B.L., Nimchinsky, E.A., and Svoboda, K. (2002). Facilitation at single synapses probed with optical quantal analysis. Nat. Neurosci. *5*, 657–664.

Partin, K.M., Patneau, D.K., Winters, C.A., Mayer, M.L., and Buonanno, A. (1993). Selective modulation of desensitization at AMPA versus kainate receptors by cyclothiazide and concanavalin A. Neuron *11*, 1069–1082.

Patneau, D.K., Vyklicky, L., Jr., and Mayer, M.L. (1993). Hippocampal neurons exhibit cyclothiazide-sensitive rapidly desensitizing responses to kainate. J. Neurosci. *13*, 3496–3509.

Rall, W. (1962). Theory of physiological properties of dendrites. Ann. N Y Acad. Sci. *96*, 1071–1092.

Regehr, W.G. (2012). Short-term presynaptic plasticity. Cold Spring Harb. Perspect. Biol. *4*, a005702.

Regehr, W.G., Carey, M.R., and Best, A.R. (2009). Activity-dependent regulation of synapses by retrograde messengers. Neuron *63*, 154–170.

Schikorski, T., and Stevens, C.F. (1997). Quantitative ultrastructural analysis of hippocampal excitatory synapses. J. Neurosci. *17*, 5858–5867.

Schiller, J., Major, G., Koester, H.J., and Schiller, Y. (2000). NMDA spikes in basal dendrites of cortical pyramidal neurons. Nature *404*, 285–289.

Spruston, N. (2008). Pyramidal neurons: dendritic structure and synaptic integration. Nat. Rev. Neurosci. *9*, 206–221.

Spruston, N., Jaffe, D.B., and Johnston, D. (1994). Dendritic attenuation of synaptic potentials and currents: the role of passive membrane properties. Trends Neurosci. *17*, 161–166.

Tsodyks, M.V., and Markram, H. (1997). The neural code between neocortical pyramidal neurons depends on neurotransmitter release probability. Proc. Natl. Acad. Sci. USA *94*, 719–723.

Varela, J.A., Sen, K., Gibson, J., Fost, J., Abbott, L.F., and Nelson, S.B. (1997). A quantitative description of short-term plasticity at excitatory synapses in layer 2/3 of rat primary visual cortex. J. Neurosci. *17*, 7926–7940.

Wadiche, J.I., and Jahr, C.E. (2001). Multivesicular release at climbing fiber-Purkinje cell synapses. Neuron *32*, 301–313.

Walker, A.S., Neves, G., Grillo, F., Jackson, R.E., Rigby, M., O'Donnell, C., Lowe, A.S., Vizcay-Barrena, G., Fleck, R.A., and Burrone, J. (2017). Distance-dependent gradient in NMDAR-driven spine calcium signals along tapering dendrites. Proc. Natl. Acad. Sci. USA *114*, E1986–E1995.

STAR★METHODS

KEY RESOURCES TABLE

REAGENT or RESOURCE	SOURCE	IDENTIFIER
Antibodies		
Rabbit polyclonal anti-VGlut1	Synaptic Systems	Cat#153303; RRID: AB_887875
Chicken polyclonal anti-GFP	Abcam	Cat# ab13970; RRID: AB_300798
Chemicals, Peptides, and Recombinant Proteins		
AP-5	Cambridge Bioscience	CAY14539; CAS: 79055-68-8
SR 95531	Cambridge Bioscience	CAY14585; CAS: 104104-50-9
QX-314	Cambridge Bioscience	CAY10011032; CAS: 24003-58-5
Alexa Fluor 594	Molecular Probes	A10438
NBQX	Santa Cruz Biotechnology	sc-222048; CAS: 479347-86-9
MK-801	Cayman Chemical Company	10009019; CAS: 77086-22-7
CTZ	Cambridge Bioscience	CAY16335; CAS: 2259-96-3
γDGG	Santa Cruz Biotechnology	sc-203728; CAS: 6729-55-1
FM4-64	Molecular Probes	T13320
CNQX	Tocris	1045; CAS: 479347-85-8
TTX	Alomone Labs	T-550; CAS: 18660-81-6
Experimental Models: Organisms/Strains		
Mouse: Syt7 KO: B6.129S1-*Syt7*^{tm1Nan}/J	The Jackson Laboratory	JAX:004950
Mouse: wt: 129S2/SvPasCrl	Charles River Laboratories	129 Mice
Oligonucleotides		
Primers: Syt7mut Forward: CTT GGG TGG AGA GGC TAT TC, Reverse: AGG TGA GAT GAC AGG AGA TC<br>Syt7wt Forward: CAT CCT CCA CTG GCC ATG AAT G Reverse: GCT TCA CCT TGG TCT CCA G	N/A	N/A
Software and Algorithms		
TrakEM2	Cardona et al., 2012	https://imagej.net/TrakEM2
Neuromorph	Jorstad et al., 2015	https://neuromorph.epfl.ch/index.html
NEURON	Hines and Carnevale, 1997	https://www.neuron.yale.edu/neuron/download

CONTACT FOR REAGENT AND RESOURCE SHARING

As Lead Contact, Juan Burrone is responsible for all reagent and resource requests. Please contact Juan Burrone at juan.burrone@kcl.ac.uk with requests and inquiries.

METHOD DETAILS

Animals

All animal procedures were approved by the local ethics committee and licensed under the UK Animals (Scientific Procedures) Act of 1986. Male and Female SV-129 mice were housed grouped in standard cages and provided with *ad libitum* food and water. The Syt 7 knockout mice were obtained from The Jackson Laboratory (Chakrabarti et al., 2003). Sprague-Dawley rats were obtained from Charles River Laboratory.

Dissociated hippocampal cultures

Primary hippocampal cultures were prepared from embryonic day 18 Sprague-Dawley rats (Charles River Laboratory). Dissociated cells were plated onto 18 mm diameter coverslips (Menzel Gläser, Germany) pre-treated with poly-D-lysine (50 μg/mL) and laminin (20 μg/mL) at a density of 350 cells/mm^2 in Neurobasal media containing 1% fetal calf serum, 1% B-27 supplement, 0.5% glutamax and 0.5% penicillin/streptomycin. Neurons were kept for 17-21 days *in vitro*.

Electron Microscopy

Two mice (post-natal day 22 and 100) were transcardially perfused with 20 mL of ice-cold saline solution followed by 200 mL of ice-cold fixative (2% PFA and 0.2% glutaraldehyde mixture in 0.1 M phosphate buffer), followed by incubation overnight in fresh fixative at 4°C. Coronal vibratome sections (60 μm) were cut using a Leica VT1000S vibratome and further fixed in 1.5% potassium ferrocyanide: 2% osmium tetroxide in cacodylate buffer for 30 min at 4°C. Tissue was then thoroughly rinsed in distilled water and incubated in 1% aqueous thiocarbohydrazide for 4 min. After further rinsing, the samples were treated with 2% aqueous osmium tetroxide for 30 min, rinsed and en-bloc stained in 1% uranyl acetate for 2 hr. To further enhance contrasts in the samples, one last treatment with Walton's Lead was carried out for 30 min at 60°C, before proceeding to dehydration in an ethanol series and infiltration with Durcupan ACM resin (Sigma). After embedding and curing, tissue blocks were mounted on Gatan 3View aluminum pins using conductive glue (CircuitWorks Conductive Epoxy) and trimmed accordingly. Before imaging, samples were gold coated to increase electron conductivity. The specimens were then placed inside a Jeol field emission scanning electron microscope (JSM-7100F) equipped with a 3View 2XP system (Gatan). Section thickness was set at 40 nm (Z resolution). Samples were imaged at 2.5kV under high vacuum using a 2048x2048 scan rate, which gave a final pixel size of 4.4 nm.

Electron microscope images were registered and manually segmented using the ImageJ plugin TrakEM2 (Cardona et al., 2012). Extracted 3D structures were exported to the Blender software with the Neuromorph toolset (Jorstad et al., 2015), which was used to compute surface, volume, and length measurements and render 3D reconstructions shown in Figure 1.

Electrophysiology

Mice (21–33 days old) were sacrificed by decapitation following Isoflurane anesthesia, the brain was immediately extracted in ice cold high sucrose solution (in mM: 240 Sucrose, 5 KCl, 1.25 Na_2PO_4, 2 $MgSO_4$, 1 $CaCl_2$, 26 $NaHCO_3$, 10 D-glucose, Saturated with 95% O_2 and 5% CO_2). In the same solution acute 300 μm thick coronal hippocampal slices were cut using a Leica vibratome (VT1000 S, Leica Microsystems). Slices were then transferred to a holding chamber containing room temperature ACSF (in mM: 125 NaCl, 5 KCl, 1.25 Na_2PO_4, 1 $MgSO_4$, 2 $CaCl_2$, 26 $NaHCO_3$, 20 D-glucose) incubated for 1 hr and kept for up to 6 hr of experiments. Cells were visualized with a Scientifica two-photon microscope equipped with a water immersion 40X 0.8 numerical aperture Olympus lens. Dodt Gradient Contrast was used to approach and patch the neurons while a Chameleon femtosecond pulsed laser (Coherent) was used for two-photon imaging of the dendritic arbors. Whole-cell recordings were performed using a Multiclamp 700B amplifier (Molecular Devices), traces were filtered at 3 KHz and digitized at 50KHz. Series resistance was < 20 MΩ. Patch pipettes were pulled (Sutter Puller P-97; Sutter Instruments) from thick-walled borosilicate glass capillaries with an inner filament (1.5 mm outer diameter, 0.86 mm inner diameter; Sutter Instruments). Pipette resistance was 3–4 MΩ after fire polishing. Voltage clamp experiments were performed in the same extracellular ACSF with the addition of 25 μM AP-5 (Cambridge Bioscience) and 20 μM SR95531 (Cambridge Bioscience). The intracellular solution contained in mM: 135 $CsMeSO_3$, 10 HEPES, 10 Na_2-Phosphocreatine, 5 Glutathione, 4 $MgCl_2$, 4 Na_2ATP, 0.4 NaGTP, 5 QX-314 (Cambridge Bioscience) and 20 μM Alexa Fluor 594 (Molecular Probes). Cells were held at −65 mV and visualized with the two-photon laser tuned at 840 nm, ≈10 min after membrane rupture to allow the dye to spread throughout. Two stimulating unipolar glass electrodes were placed in the distal and proximal dendritic region. Stimulus intensity ranged between 0.1 and 0.8 mA using an Iso-Flex stimulator (Intracel). Fibers were stimulated 20 times with 5 pulse trains every 30 s to calculate the average EPSC. For the NMDAR depletion experiments the extracellular solution contained the AMPAR channel blocker NBQX 10 μM (Santa Cruz Biotechnology) instead of AP-5. Baseline recordings of synaptic inputs, stimulated at proximal and distal sites, were obtained as above but with a single stimulation pulse repeated 20 times every 10 s. MK-801(40 μM, Cayman Chemical Company) was bath applied, and allowed to equilibrate for 5 min, after which the stimulation of either the proximal or distal site was repeated a further 40 times to obtain the curves in Figure 2J. For cross-depletion experiments in Figure S3, we continued the experiment and delivered another 40 stimuli to the site that had not been depleted. Care was taken to vary the site (proximal or distal) that was depleted first. In current clamp experiments the extracellular ACSF contained 20 μM SR95531 while the intracellular solution contained in mM: 115 K-$MeSO_4$, 20 KCl, 10 Na_2-Phosphocreatine, 10 HEPES, 2 MgATP, 2 Na_2ATP, 0.4 mM Na_2GTP, and 20 μM Alexa Fluor 594. The stimulus was delivered with a single bipolar glass theta electrode either distally or proximally with intensity ranging between 1 and 6.2 V. All recordings were performed at 30°C. Fibers were stimulated every 1 min with increasing voltage intensity consisting of 0.2 V steps. All electrophysiology experiments were analyzed in IGOR Pro software (Wavemetrics) with the NeuroMatic 2.7 package and MATLAB (MathWorks). To detect non-linear events, a linear fit was calculated for the gradual increase in EPSP amplitude in response to increasing current steps of the first pulse. The expected amplitude of the second pulse was calculated from this fit. If the recorded EPSP was 2 V above the expected amplitude it was then classified as a non-linearity. All chemicals were from Sigma unless otherwise stated.

Modeling

Simulations were performed with the NEURON simulation environment (Hines and Carnevale, 1997). The model consisted of a soma connected to one dendrite with length of one length constant distributed over 50 segments (diameter = 1 μm). Passive parameters were Cm = 1 μF/cm2, Rm = 10,000 Ω.cm2, Ra = 80 Ω·cm and a leak conductance with a reversal of −65 mV. Unless otherwise noted, active conductances in the dendrite were (in mS/cm2): voltage-activated sodium channels (4), voltage-activated potassium channels (0.8), M-type potassium channels (0.005), high-threshold voltage-activated calcium channels (0.05), low-threshold voltage-activated calcium channels (0.15 × 10-3). Fifteen synapses containing AMPA and NMDA receptor conductances were distributed uniformly

over the first or last 50% of the dendrite for the proximal and distal scenarios, respectively. Synaptic conductances were modeled as double exponential functions with gmax = 0.5 nS, $\tau 1$ = 0.1 ms, $\tau 2$ = 1 ms for AMPA and $\tau 1$ = 1 ms, $\tau 2$ = 20 ms for NMDA. For fitting short-term plasticity dynamics we initially used the Tsodyks and Markram model (Tsodyks and Markram, 1997), and fitted it by systematically varying the model parameters and comparing the model fits using the peak postsynaptic conductance for each presynaptic pulse on a train of 5 pulses according to $\sum_{i=1}^{5}|modelG_i - dataG_i|$, where $modelG_i$ and $dataG_i$ are the peak postsynaptic conductance for model and data respectively, for pulse *i*. While this model produced good fits for the first half of the pulse train, it failed to satisfactorily capture the profile of the remaining pulses. We then fitted the Varela et al. model (Varela et al., 1997) following the same procedure, which produced a good fit to all 5 pulses, mainly because of separately accounting for fast and slow depression kinetics. The Varela et al. model (Varela et al., 1997) was then used in all simulations. Short-term plasticity was implemented with time constants adjusted to match the experimental data and applied to both AMPA and NMDA conductances (facilitation: 2.5 distal, 1.6 proximal; fast depression: 0.4; slow depression: 0.94).

For the passive scenario, active conductances were turned off and synapses only contained AMPA conductances. For the data shown in Figures 3A and 3B a single synapse was activated with a train of ten pulses at 5-100 Hz, and in Figures 3C–3E independent Poisson trains 500 ms long were delivered at each synapse with frequencies varying from 5-80 Hz. Mean depolarization and standard deviation in Figures 3D and 3E were measured during the stimulation period. All simulations were performed at 35°C. For the model variations shown in Figure S4, the following variables were changed for distal synapses: release probability was reduced by 30%, AMPA conductance was reduced by 19%, and dendrite diameter was reduced by 15% (all of the values were estimated from experimental data). The decrease in mean P_r was taken from the double exponential fit in Figure 2J, where the time-constant is inversely proportional to P_r (Hessler et al., 1993). We took the mean time-constant for a double exponential fit, taking into account the fraction of the curve each one accounted for ($\tau_{mean} = \tau_{fast} * fr_{fast} + \tau_{slow} * fr_{slow}$, where τ_{fast} and τ_{slow} are the fast and slow time-constants and fr_{fast} and fr_{slow} are the fractions of the curve they account for), and calculated the change in τ_{mean} (proximal: 8.09 ms; distal: 11.49 ms), which is inversely proportional to mean P_r. We find that distal synapses show a ~30% decrease in mean P_r when compared to proximal compartments. The code for the mathematical model is provided as supplementary information (see supplementary text).

Immuno-histochemistry and FM4-64 staining of primary neurons

Dissociated hippocampal neurons expressing GCaMP3 were immunostained using the following primary antibodies: rabbit αVGlut1 (1:500 Synaptic Systems) and chicken αGFP (1:1000, Abcam). Neurons were fixed in 4% PFA for 20 min and permeabilized using 0.25% Triton X-100 (Sigma, UK) in PBS for 5 min. Cells were incubated with 10% goat serum (Sigma, UK) for 1 hr at room temperature, then incubated with primary antibodies in 2% goat serum overnight at 4°C, and finally with Alexa-conjugated secondary antibodies (1:1000, Molecular Probes) for 1 hr at room temperature. Coverslips were mounted onto glass microscope slides using mowiol. Imaging of immunostained neurons was performed using an Olympus FV1000 confocal microscope equipped with a 40X/0.8 NA water-immersion objective (Olympus).

FM4-64 staining was performed by incubating neurons in high K+ HBS (78.5mM NaCl, 60mM KCl, 10mM HEPES, 10mM Glucose, 2mM CaCl, 1.3mM MgCl) supplemented with 10 μM FM4-64 (Molecular Probes), 1 μM TTX (Alamone Labs), 2.5nM AP-5 and 2nM CNQX (Tocris) for 90 s to load the entire releasable pool of vesicles. Cells were then washed twice for 4 min in HBS (139mM NaCl, 2.5mM KCl, 10mM HEPES, 10mM Glucose, 2mM CaCl, 1.3mM MgCl, 1 μM TTX) to remove FM4-64 from all external membranes. Neurons were then imaged using an Olympus IX71 inverted microscope with a CCD camera (Coolsnap HQ) controlled by Slidebook software (Intelligent Imaging Innovations), equipped with a 40X/1.0 NA oil-immersion objective (Olympus). The excitation light source was a xenon-arc lamp (Lambda LS; Sutter Instruments), in which light exposure was regulated by a rapid shutter (smart-Shutter; Sutter Instruments) controlled by a Sutter Instruments lambda 10-3 controller. Filtering was provided by a 470 ± 20 nm band pass excitation and 515 ± 20 nm band pass emission (Chroma Technology Corporation) filter set for GCaMP3 and a 565 ± 22 nm band pass excitation and 590-nm long pass dichroic plus 650 ± 36 nm band pass emission (Chroma Technology Corporation) filters for FM4-64.

Statistics

Statistical analysis was performed in MATLAB (MathWorks) and Prism (Graphpad), all the data analyzed with parametric tests was first tested for normality with the D'Agostino and Pearson normality test.

Further information and requests for resources and reagents should be directed to and will be fulfilled by the Lead Contact, Juan Burrone (juan.burrone@kcl.ac.uk).

Neuron, Volume *99*

Supplemental Information

A Distance-Dependent Distribution of Presynaptic Boutons Tunes Frequency-Dependent Dendritic Integration

Federico W. Grillo, Guilherme Neves, Alison Walker, Gema Vizcay-Barrena, Roland A. Fleck, Tiago Branco, and Juan Burrone

Figure S1

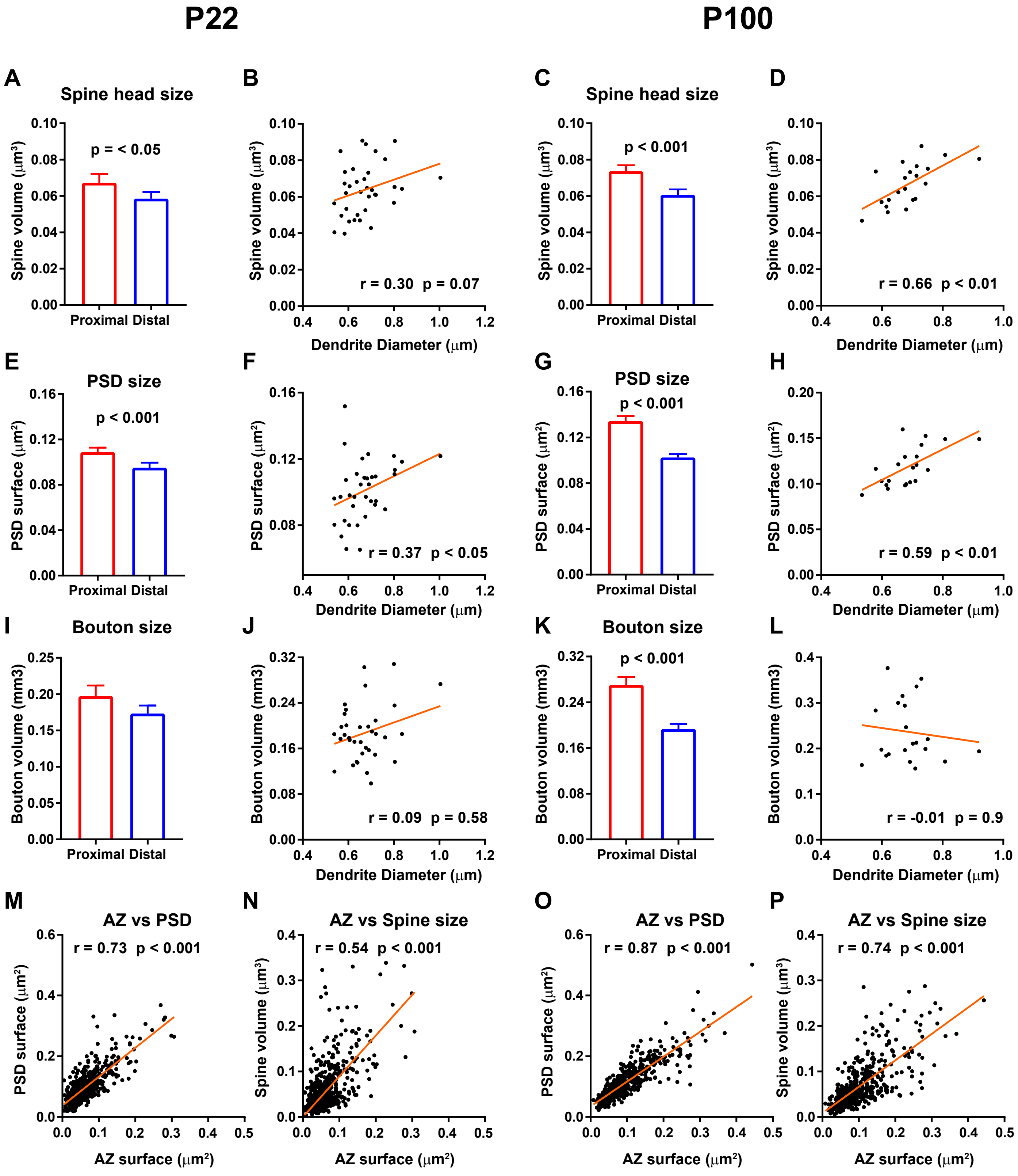

P22
P100
A
Spine head size
Spine volume (µm³)
p = < 0.05
Proximal Distal
B
Spine volume (µm³)
r = 0.30 p = 0.07
Dendrite Diameter (µm)
C
Spine head size
Spine volume (µm³)
p < 0.001
Proximal Distal
D
Spine volume (µm³)
r = 0.66 p < 0.01
Dendrite Diameter (µm)
E
PSD size
PSD surface (µm²)
p < 0.001
Proximal Distal
F
PSD surface (µm²)
r = 0.37 p < 0.05
Dendrite Diameter (µm)
G
PSD size
PSD surface (µm²)
p < 0.001
Proximal Distal
H
PSD surface (µm²)
r = 0.59 p < 0.01
Dendrite Diameter (µm)
I
Bouton size
Bouton volume (mm3)
Proximal Distal
J
Bouton volume (mm3)
r = 0.09 p = 0.58
Dendrite Diameter (µm)
K
Bouton size
Bouton volume (mm3)
p < 0.001
Proximal Distal
L
Bouton volume (mm3)
r = -0.01 p = 0.9
Dendrite Diameter (µm)
M
AZ vs PSD
r = 0.73 p < 0.001
PSD surface (µm²)
AZ surface (µm²)
N
AZ vs Spine size
r = 0.54 p < 0.001
Spine volume (µm³)
AZ surface (µm²)
O
AZ vs PSD
r = 0.87 p < 0.001
PSD surface (µm²)
AZ surface (µm²)
P
AZ vs Spine size
r = 0.74 p < 0.001
Spine volume (µm³)
AZ surface (µm²)

Figure S1. Synaptic properties for P22 and P100 dendrites reconstructed by SBFSEM. Related to Figure 1. For all P22 mice, N = 35 dendrites, 604 synapses. For P100 mice, N = 26 dendrites, 505 synapses. **(A-D)** Postsynaptic spine volume is smaller in distal dendrites compared to proximal dendrites in P22 and P100 mice (Wilkoxon Rank-Sum for A and C; Spearman's correlation for B and D). **(E-H)** Postsynaptic density (PSD) is smaller in distal dendrites compared to proximal dendrites in P22 and P100 mice (Wilkoxon Rank-Sum for E and G; Spearman's correlation for F and H). **(I-L)** Presynaptic bouton size (volume) in distal dendrites compared to proximal dendrites in P22 and P100 mice (Wilkoxon Rank-Sum for I and K; Spearman's correlation for J and L). **(M-P)** Correlations between PSD and AZ size (M and O, Spearman's correlation) and between spine volume and AZ size (N and P, Spearman's correlation) in P22 and P100 mice.

Figure S2

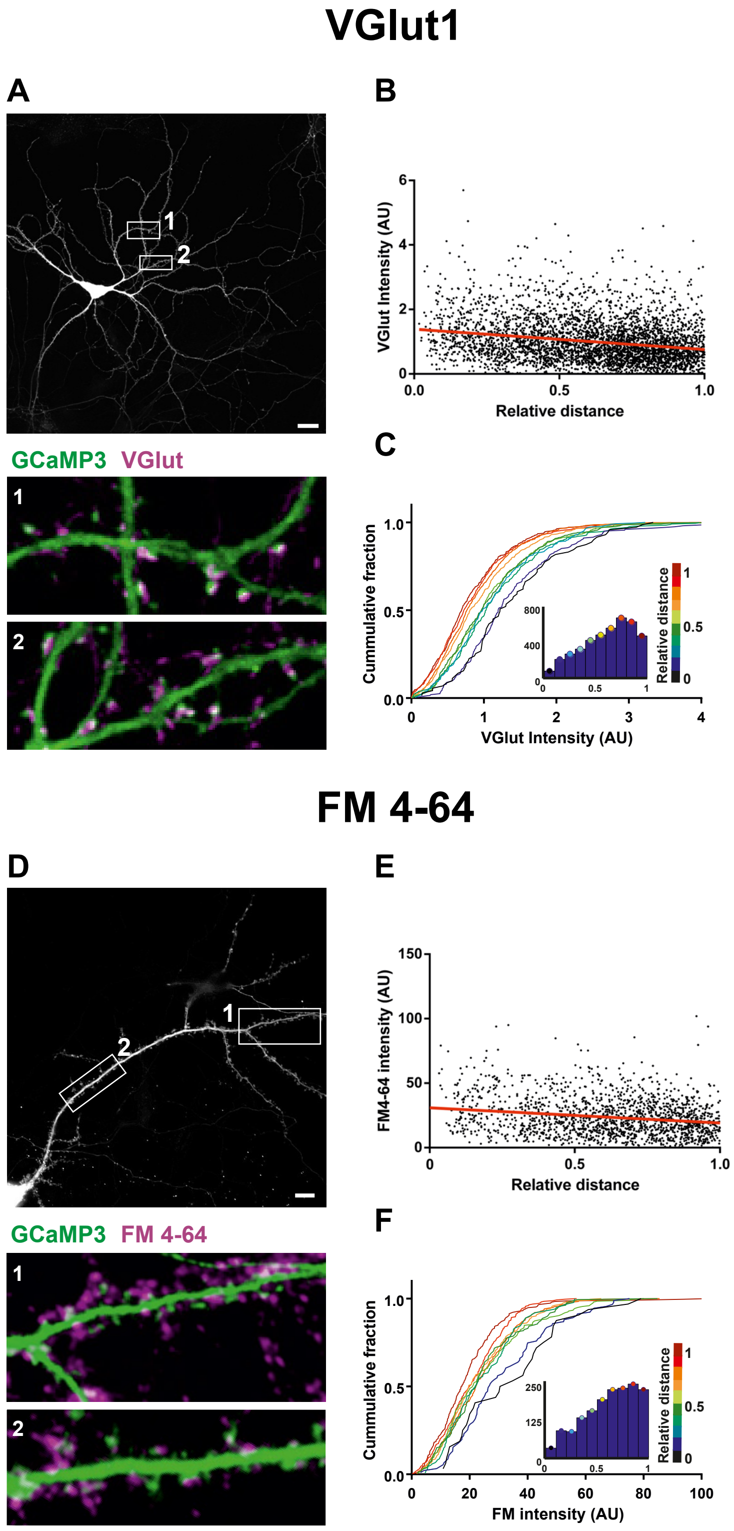
VGlut1
A
B
1
2
VGlut Intensity (AU)
Relative distance
C
GCaMP3 VGlut
Cummulative fraction
VGlut Intensity (AU)
Relative distance
FM 4-64
D
E
FM4-64 intensity (AU)
Relative distance
F
GCaMP3 FM 4-64
Cummulative fraction
FM intensity (AU)
Relative distance

Figure S2. Preynaptic structure and function scales inversely with relative distance along a dendrite in dissociated neurons. Related to Figure 2. (A-C) Immunocytochemical labelling of vGlut1. **(A)** Example hippocampal neurons expressing GCaMP3, used here as a cytoplasmic marker. White box corresponds to the areas shown in the zoomed region below, numbered appropriately. Scale bars = 10 µm. Zoomed regions show immunostain (magenta) overlaid onto the GCaMP3 expressing cell (green). **(B)** vGlut immunostain intensity for each puncta plotted as a function of relative dendritic distance to the soma. Spearman's correlation ($P<0.0001$, $R^2=0.94$, n=4051 from 4 cells). **(C)** Cumulative frequency distributions for immunostain intensity for each pucta where each plot is colour-matched to a different bin for relative distance shown in the key to the right. Inset histograms show the number of spines included within each relative distance bin. **(D-F)** Labelling of vesicle recylcling with FM4-64. **(D)** Example hippocampal neurons expressing GCaMP3. White box corresponds to the areas shown in the zoomed region below, numbered appropriately. Scale bars = 10 µm. Zoomed regions show FM4-64 stain (magenta) overlaid onto the GCaMP3 expressing cell (green). **(E)** FM4-64 label intensity for each puncta plotted as a function of relative dendritic distance to the soma. Spearman's correlation ($P=0.001$, $R^2=0.85$, n=1650 spines from 7 cells). **(F)** Cumulative frequency distributions for FM4-64 intensity for each pucta where each plot is colour-matched to a different bin for relative distance shown in the key to the right. Inset histograms show the number of spines included within each relative distance bin. Relative dendritic distance was calculated as the absolute distance (shortest path to the soma) divided by the distance from the soma to the furthest branch tip, calculated using an algorithm that searches for the longest path from each ROI to a branch tip.

Figure S3

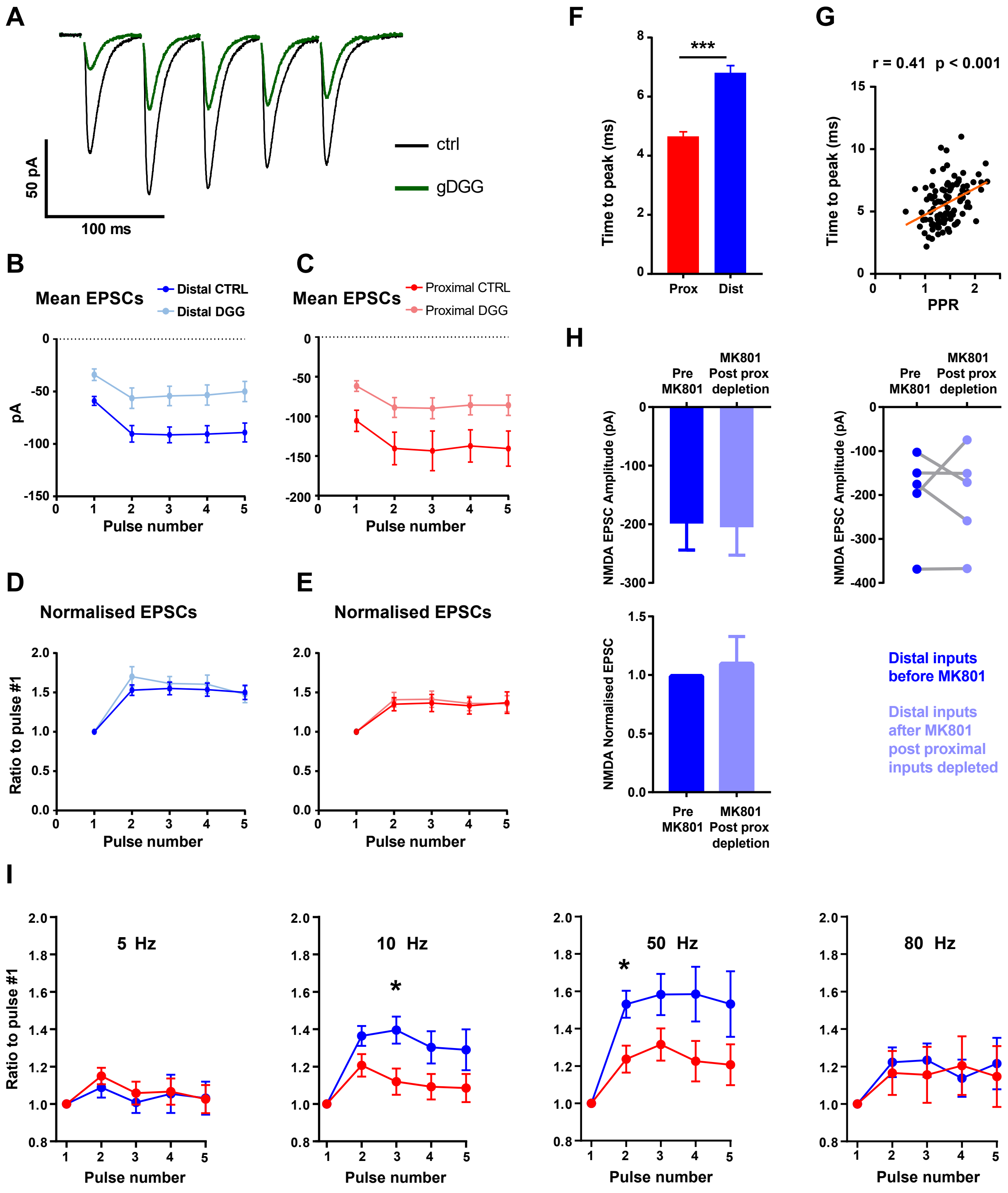
A
50 pA
100 ms
ctrl
gDGG
B
Mean EPSCs
Distal CTRL
Distal DGG
pA
Pulse number
C
Mean EPSCs
Proximal CTRL
Proximal DGG
Pulse number
D
Normalised EPSCs
Ratio to pulse #1
Pulse number
E
Normalised EPSCs
Pulse number
F
***
Time to peak (ms)
Prox
Dist
G
r = 0.41 p < 0.001
Time to peak (ms)
PPR
H
Pre MK801
MK801 Post prox depletion
NMDA EPSC Amplitude (pA)
NMDA Normalised EPSC
Distal inputs before MK801
Distal inputs after MK801 post proximal inputs depleted
I
5 Hz
10 Hz
50 Hz
80 Hz
*
Ratio to pulse #1
Pulse number

Figure S3. **Physiological properties of proximal and distal synapses. Related to Figure 2.** γ-D-Glutamylglycine (γDGG) is an AMPA glutamate receptor antagonist with low affinity and fast dissociation kinetics. When present in the millimolar range it competes with released glutamate causing a reduction of AMPAR current. **(A)** EPSC amplitude is reduced, compared to control (black trace), following γDGG application (gray trace). **(B-C)** Mean 5 pulse stimulus response amplitudes before and during 1.5 mM γDGG bath application. **(D-E)** Normalised EPSCs for 5 pulse trains before and during 1.5 mM γDGG bath application. Although not significant ($P = 0.15$, two-tailed paired t test) there is a small increase in PPR that is more marked in distal **(D)** compared to proximal synapses **(E)** and may reflect an increased occurrence of multi vesicular release between the first and second pulse. **(F)** The time to peak of an EPSC was significantly higher in distally triggered events (49 cells, mean = prox 6.8 ± 0.24 ms, dist 4.65 ± 0.16 ms, $p < 0.0001$ Wilcoxon sign rank test) with longer times correlating to the amount of facilitation **(G)**, Spearman's correlation. **(H)** Distal NMDAR mediated responses are not affected following blockade of proximal NMDARs with MK-801, N=5 cells. Top left: average amplitude of EPSCs prior to MK801 bath application (dark blue, average of 20 sweeps) and after proximal inputs have been depleted (First response to firs stimulus) in MK-801 stimulation protocol (Figure 2J) (light blue); top right: changes in the amplitudes of proximal NMDAR responses for individual cells following depletion of distal NMDAR responses (N = 5 cells); bottom left: average NMDAR responses normalized to the response before MK-801 proximal depletion. **(I)** Facilitation over trains of 5 pulses is frequency-dependent. No statistical difference between proximal and distal responses found at 5Hz (n = 13 cells) and 80 Hz (n = 9). At 10 Hz (n = 16) greater distal facilitation was detected at pulse #3, $p < 0.05$; at 50 Hz (n = 20) greater distal facilitation was detected at pulse #2, $p < 0.05$, multiple t tests with Holm-Sidak adjusted p values.

Figure S4

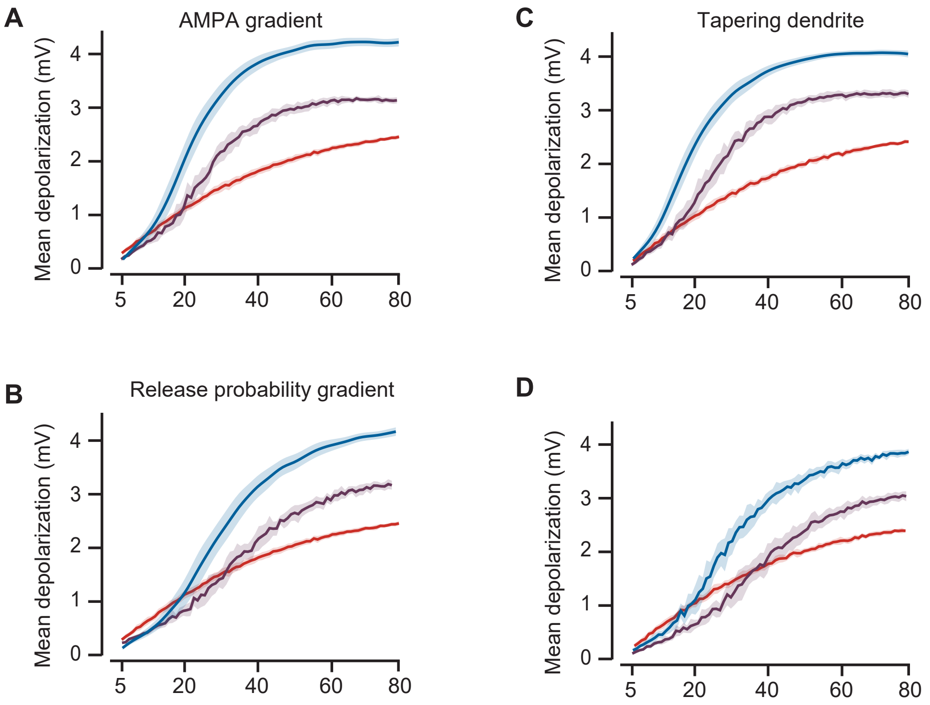

A
AMPA gradient
B
Release probability gradient
C
Tapering dendrite
D
Mean depolarization (mV)
0
1
2
3
4
5
20
40
60
80

Figure S4. PPR gradient enhances distal supra-linear integration in the presence of additional gradients. Related to Figure 3. Input-output curves for models with proximo-distal gradients of **(A)** AMPA receptor conductances (distal = 81% of proximal), **(B)** release probability (distal = 70% of proximal), **(C)** dendrite diameter (distal = 85% of proximal), and **(D)** all three gradients together. For all plots red curves show proximal synapses, blue show distal synapses and purple show distal synapses with proximal PPR. Shaded area is SEM. All values for the gradients used in the model were obtained from measurements taken from Figures 1 and 2.

Supplementary text. Code for the compartmental model used in Figure 3 and S4. Compartmental model of dendritic integration that implements synaptic short-term facilitation and depression as described previously (Varela et al., 1997). Simulations were performed with the NEURON simulation environment (Hines and Carnevale, 1997).
